# fMRI-based validation of continuous-wave fNIRS of supplementary motor area activation during motor execution and motor imagery

**DOI:** 10.1038/s41598-022-06519-7

**Published:** 2022-03-04

**Authors:** Franziska Klein, Stefan Debener, Karsten Witt, Cornelia Kranczioch

**Affiliations:** 1 Neurocognition and Neurorehabilitation Group, Department of Psychology, School of Medicine and Health Sciences, University of Oldenburg, Oldenburg, Germany; 2 Neuropsychology Lab, Department of Psychology, School of Medicine and Health Sciences, University of Oldenburg, Oldenburg, Germany; 3 Neurology, Department of Human Medicine, School of Medicine and Health Sciences, University of Oldenburg, Oldenburg, Germany

**Keywords:** Neuroscience, Psychology

## Abstract

Compared to functional magnetic resonance imaging (fMRI), functional near infrared spectroscopy (fNIRS) has several advantages that make it particularly interesting for neurofeedback (NFB). A pre-requisite for NFB applications is that with fNIRS, signals from the brain region of interest can be measured. This study focused on the supplementary motor area (SMA). Healthy older participants (N = 16) completed separate continuous-wave (CW-) fNIRS and (f)MRI sessions. Data were collected for executed and imagined hand movements (motor imagery, MI), and for MI of whole body movements. Individual anatomical data were used to (i) define the regions of interest for fMRI analysis, to (ii) extract the fMRI BOLD response from the cortical regions corresponding to the fNIRS channels, and (iii) to select fNIRS channels. Concentration changes in oxygenated ($\Delta [HbO]$) and deoxygenated ($\Delta [HbR]$) hemoglobin were considered in the analyses. Results revealed subtle differences between the different MI tasks, indicating that for whole body MI movements as well as for MI of hand movements $\Delta [HbR]$ is the more specific signal. Selection of the fNIRS channel set based on individual anatomy did not improve the results. Overall, the study indicates that in terms of spatial specificity and task sensitivity SMA activation can be reliably measured with CW-fNIRS.

## Introduction

Functional near infrared spectroscopy (fNIRS) has gained considerable popularity over the past decades. This popularity is to a large part owed to the fact that compared to the gold standard for in vivo imaging of the brain, functional magnetic resonance imaging (fMRI), less restrictions and safety concerns apply to fNIRS. fNIRS devices range from transportable to portable and even wireless, allowing greater flexibility regarding participant behaviour and types of experimental settings^1,2^.

Similar to fMRI, fNIRS captures hemodynamic changes. This is achieved by placing optodes consisting of light sources and light detectors on the head surface. Near infrared light is transmitted into the tissue between source and detector and hemoglobin absorption can be quantified as concentration changes in oxygenated ($\Delta [HbO]$) and deoxygenated ($\Delta [HbR]$) hemoglobin^2,3^. Unlike fMRI, fNIRS has no environmental restrictions and no contraindications, is less expensive, has been shown to tolerate motion, and it has a higher temporal resolution^4^.

One important limitation of fNIRS is the modest depth penetration of near infrared light. Therefore fNIRS does not capture subcortical activation and is limited to superficial cortical brain regions^4^. Another limitation is that optode placement and data processing pipelines have to cope with a lack of anatomical information^4^. Because of the lacking anatomical information, correct placement of the available optodes is important and of utmost relevance if the goal is to collect data from a particular region of interest (ROIs). Software has been developed to address this issue (e.g., fOLD^5^ and AtlasViewer^6^) by guiding optode placement using anatomical information taken from standard brains. While this approach is without doubt very useful, the selection of channels for subsequent analysis may benefit from the consideration of individual anatomical information. Finally, a third limitation is the relatively low fNIRS signal-to-noise ratio, largely resulting from contamination by systemic noise (i.e., non-neuronal evoked and non-evoked physiological processes^3,7^). Demands for noise attenuation based on statistical procedures^8–10^ were recently met by making the necessary hardware-based solutions for dealing with physiological noise available^11,12^.

Despite these challenges, fNIRS can be easily applied repeatedly and is therefore an excellent technology for brain computer interfaces (BCIs^1^), neurofeedback research and application^13^. In motor-related neurorehabilitation, individuals learn to self-regulate motor areas by receiving feedback based on task-related brain activations^13,14^. This may facilitate cortical reorganization that is necessary to initiate compensation and recovery. In most motor neurofeedback and motor BCI applications, brain activation is generated by asking users to imagine movements. A mental simulation of the sensation of a movement^15,16^ is known as kinesthetic motor imagery (MI). FNIRS MI neurofeedback has been used with encouraging results for the support of motor recovery in stroke patients^17,18^. A relatively new, unexplored field for fNIRS MI neurofeedback is the alleviation of motor symptoms in patients suffering from Parkinson’s disease (PD). In two fMRI studies^15,19^ patients imagined whole-body movements while receiving neurofeedback from the supplementary motor area (SMA) in a small number of sessions. In addition to subcortical brain areas affected in PD, the SMA is known to be highly underactive in these individuals^20,21^. A systematic SMA upregulation training has been proposed to produce lasting changes of the cortico-basal ganglia-circuit and to drive symptom improvements^15,19^. In order to pave the way towards a future fNIRS SMA upregulation neurofeedback training protocol, we here investigated whether fNIRS reliably captures motor execution and motor imagery induced SMA activation.

A number of studies have explored motor execution (ME) induced primary motor (M1) activation with simultaneous^22–29^ and consecutive fNIRS-fMRI setups^30^. We are aware of only one study exploring the sensitivity of fNIRS for capturing MI-induced brain activity from SMA and premotor cortex^31^. The authors used laser-based time-resolved fNIRS (TR-fNIRS) rather than the more common diode-based continuous wave fNIRS (CW-fNIRS) technology, arguing that TR-fNIRS might provide better depth sensitivity. A main finding was that TR-fNIRS detected MI-related brain activity in SMA. Whether the same holds for CW-fNIRS has not been shown yet. The present study aims at closing this gap by considering spatial specificity and task sensitivity properties of SMA activity measured by CW-fNIRS. A second aim of this study was to contribute information regarding the choice of the signal used for CW-fNIRS neurofeedback, that is, $\Delta [HbO]$ or $\Delta [HbR]$. Most previous fNIRS neurofeedback studies employed $\Delta [HbO]$ signals of motor areas because of larger amplitudes as compared to $\Delta [HbR]$ signals^13^. When compared to fMRI, there is no agreement on whether $\Delta [HbO]$ or $\Delta [HbR]$ provide better spatial specificity and task sensitivity^4^. Finally, a third aim of this study was to assess whether individual anatomical images are needed to improve fNIRS channel selection and thereby future fNIRS neurofeedback application.

In the present study, (f)MRI and fNIRS data were collected in a consecutive setup. The main region of interest was the SMA, for which both ME and MI data were analysed. Bilateral M1 was included for the purpose of validation of the overall procedure. M1 analyses were restricted to ME data. For ME and M1 it was expected that analyses would confirm that ME results in activation in CW-fNIRS channels in spatial correspondence to M1 fMRI activation, with M1 defined by individual anatomical images, and that the M1 fNIRS signal follows a similar time course as the M1 fMRI signal (spatial specificity). Comparable task-related modulations were expected for fNIRS and fMRI, in particular a lateralisation of activation with stronger activation in contralateral M1 (task sensitivity). For SMA it was predicted that ME and MI activate SMA as confirmed by (f)MRI, and that the activation is evident in spatially corresponding fNIRS channels. The time course of SMA fNIRS data was expected to match the SMA fMRI time course (spatial specificity). Further, task-related differences in fMRI SMA activation were predicted to be evident also in fNIRS SMA data, in particular a stronger activation for ME than MI task was expected (task sensitivity). To address the choice of neurofeedback signal, spatial specificity and task sensitivity were considered for both, $\Delta [HbO]$ and $\Delta [HbR]$. Regarding the value of individual anatomical images for fNIRS analysis, analyses were performed twice, once with the full fNIRS channel set and once with channels that were selected based on individual anatomy.

## Results

### Subjects

Out of 34 participants (17 females, 17 males) a total of 18 participants were excluded because of the following reasons: mild cognitive decline as detected by the MoCa (2), poor fNIRS signal quality (2), falling asleep in the MR scanner (1), typing with the wrong hand (2), forgetting repeatedly not to execute the movements during MI (1), cancelling the second session (1), insufficient cap placement (2), a beta mask that did not cover the whole cortex (1), and electromyography (EMG) signal saturation (fMRI session) (2) and excessive movement during MI (4). The latter was the case if for a participant more than half of the trials of a single MI task were classified as movement trials in either of both sessions (fNIRS or fMRI; for more details cf. section *Electromyography (EMG)*). A sample of N = 16 subjects (10 females, 6 males; age [mean ± SD]: [64.00 ± 5.27] years; range 56 to 71 years) remained for analyses.

### Spatial specificity

#### Between subjects: topographical similarity

**Figure 1:**
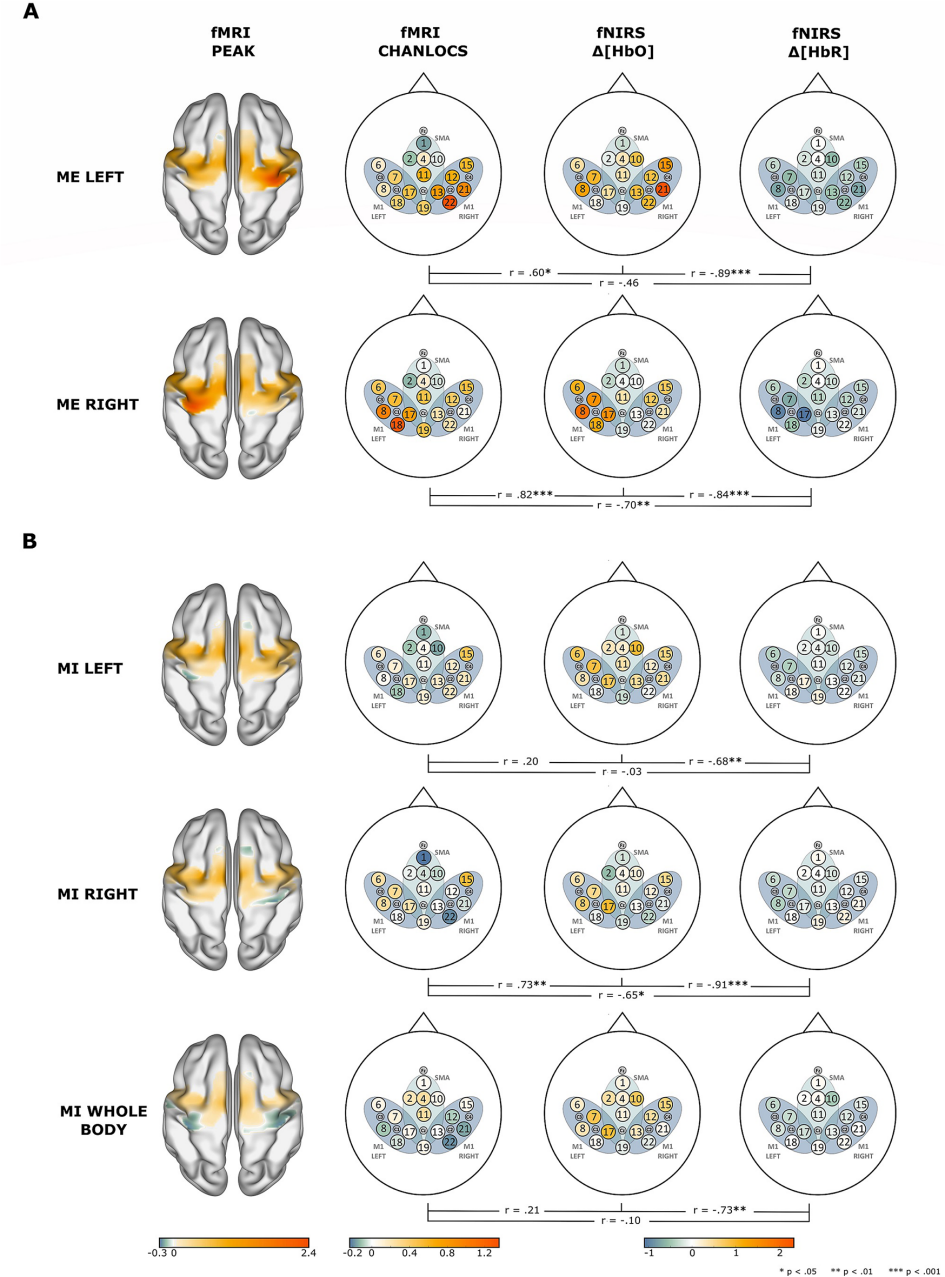
Between subjects: topographical similarity between data types fMRI CHANLOCS, fNIRS $\Delta [HbO]$, and fNIRS $\Delta [HbR]$ of **(A)** ME tasks and **(B)** MI tasks. For comparison, fMRI PEAK maps are included in the first column. Note that beta color bars are different for fMRI PEAK, fMRI CHANLOCS, and fNIRS data types. Lines below the maps show the Spearman correlation coefficients of the correlation between the respecting two connected topographic maps. Beta maps of fMRI PEAK were visualized using BrainNet Viewer^32^ (http://www.nitrc.org/projects/bnv/).

Figure 1 illustrates the averaged beta topographic maps for all tasks and data types (fMRI PEAK, fMRI CHANLOCS, fNIRS $\Delta [HbO]$ and fNIRS $\Delta [HbR]$). Descriptively, regarding the ME data (cf. Fig. 1A), for both ME LEFT and ME RIGHT a clear M1 lateralisation with comparable spatial patterns can be seen for fMRI PEAK and fMRI CHANLOCS data. For fNIRS $\Delta [HbO]$ and fNIRS $\Delta [HbR]$ data types this lateralisation seems reduced for ME LEFT for the fNIRS $\Delta [HbR]$ data type. However, the spatial patterns of fNIRS $\Delta [HbO]$ and fNIRS $\Delta [HbR]$ data are very similar. For the MI tasks (cf. Fig. 1B) the spatial patterns of fMRI CHANLOCS data type and fNIRS data types seem less comparable among one another. However, fNIRS $\Delta [HbO]$ and fNIRS $\Delta [HbR]$ data show similar spatial patterns. Descriptively, the MI WHOLE BODY task seems to be the most spatially specific within the ROI SMA.

Spearman correlations were performed between the averaged beta maps of data types fMRI CHANLOCS, fNIRS $\Delta [HbO]$ and fNIRS $\Delta [HbR]$ to test for topographical similarity. For ME LEFT and ME RIGHT all correlations were significant ($p < .05$; cf. Fig. 1A for exact values), except for the comparison between fMRI CHANLOCS and fNIRS $\Delta [HbR]$ for task ME RIGHT. For the MI tasks the picture was slightly mixed. For MI RIGHT all correlations were significant for all pairs (all $p < .05$; cf. Fig. 1B for exact values). For MI LEFT and MI WHOLE BODY only the comparisons between fNIRS $\Delta [HbO]$ and fNIRS $\Delta [HbR]$ were significant ($p < .01$). Overall, these results indicate a high spatial specificity for the fNIRS data types of the ME tasks. Spatial specificity of the fNIRS data type is somewhat reduced in the MI tasks.

#### Within subjects: time series correlation

**Figure 2:**
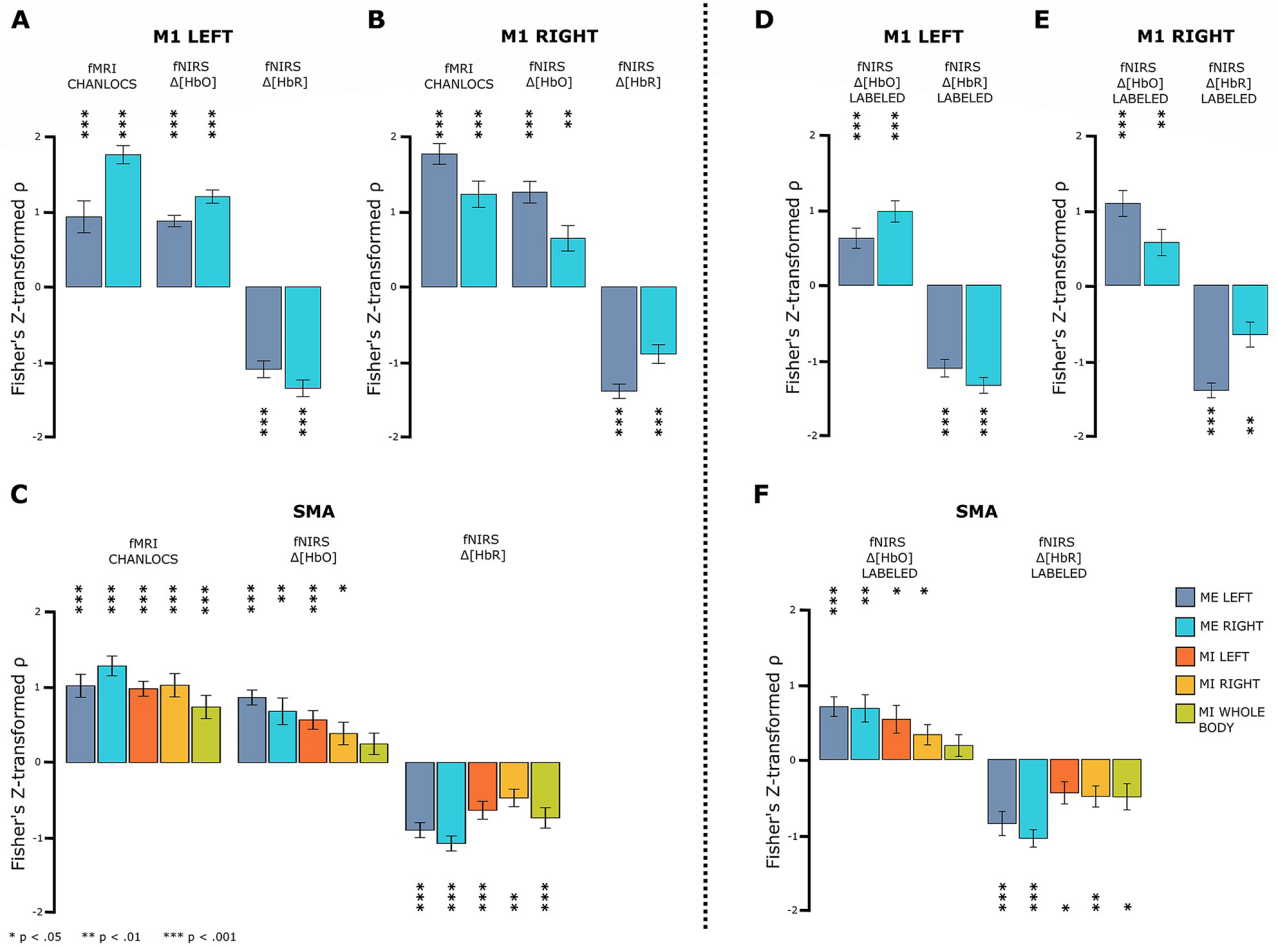
Within subjects: time series correlation. **(A,B)** Bar plots of the averaged Fisher’s z-transformed Spearman correlation coefficients between the individual grand average time series data of fMRI PEAK data type and data types fMRI CHANLOCS, fNIRS $\Delta [HbO]$ and fNIRS $\Delta [HbR]$ for ME tasks. **(D,E)** Illustrate the same for fNIRS $\Delta [HbO]$ and fNIRS $\Delta [HbR]$ LABELED. In **(C)** SMA data are shown for all tasks and data types and in **(F)** the same for fNIRS $\Delta [HbO]$ and fNIRS $\Delta [HbR]$ LABELED. Note: Error bars represent standard error of the mean. Asteriscs indicate statistical significance.

Within subject analysis of spatial specificity focused on time series correlations within ROIs. Individual grand average time series data of fMRI PEAK data type and all other data types were Spearman correlated separately for each ROI. As illustrated in Fig. 2, the averaged Fisher’s z-transformed Spearman correlation coefficients were generally positive for fMRI CHANLOCS and fNIRS $\Delta [HbO]$ and negative for fNIRS $\Delta [HbR]$.

The results of one-sample t-tests indicated for all data types a strong relationship with the fMRI PEAK data type for the ME tasks in the M1 ROIs (all $p < .01$, all $|d| \ge 0.95$). In addition, all analyses revealed a stronger correlation in the hemisphere contralateral to the executed hand. Patterns and statistical results were virtually identical for LABELED fNIRS data. (cf. Fig.  2A,B,D,E and Table 1).

For ROI SMA (cf. Fig.  2C,F), the pattern was less homogeneous. For both ME tasks t-tests were likewise highly significant for all data types (all $p < .05$, all $|d| \ge 0.96$; cf. Table 2). For the MI tasks all t-tests were highly significant for fMRI CHANLOCS (all $p < .001$, all $|d| > 1.19$; cf. Table 2). For fNIRS $\Delta [HbR]$ all MI tasks ($p < .01$, $|d| \ge 1.01$) and for fNIRS $\Delta [HbO]$ MI LEFT and MI RIGHT tasks ($p < .05$, $|d| \ge 0.64$) were significantly different from zero.

In sum, the results confirmed the predicted task-related spatial specificity of fNIRS time series data, most clearly for ME tasks. For the MI tasks spatial specificity was strongest in $\Delta [HbO]$ for MI LEFT and for $\Delta [HbR]$ in MI WHOLE BODY.

##### Channel labeling

Descriptively, for the vast majority of combinations of ROI, task and data type, statistical values and effect sizes decreased for the LABELED fNIRS data types (cf. Tables 1 and 2). The only exceptions were the combination of ME RIGHT and $\Delta [HbR]$ LABELED at ROI M1 LEFT as well as ME LEFT and $\Delta [HbO]$ LABELED at ROI SMA.

**Table 1:** Within subjects: time series correlation (ME tasks and M1 ROIs).

fMRI PEAK $\sim$	ME LEFT					
	M1 LEFT			M1 RIGHT		
	fMRI CHANLOCS	fNIRS $\Delta [HbO]$	fNIRS $\Delta [HbR]$	fMRI CHANLOCS	fNIRS $\Delta [HbO]$	fNIRS $\Delta [HbR]$
(mean ± SEM)	($0.93\pm 0.21$)	($0.88\pm 0.08$)	($-1.10\pm 0.11$)	($1.77\pm 0.14$)	($1.26\pm 0.14$)	($-1.39\pm 0.09$)
t-test	$$\begin{array}{l}t(15) = 4.40\\ \varvec{p < .001}\\ d = 1.10\\ 95\%CI [0.48, 1.38]\end{array}$$	$$\begin{array}{l}t(15) = 11.44\\ \varvec{p < .001}\\ d = 2.86\\ 95\%CI [0.71, 1.04]\end{array}$$	$$\begin{array}{l}t(15) = -9.82\\ \varvec{p < .001}\\ d = -2.45\\ 95\%CI [-1.34, -0.86]\end{array}$$	$$\begin{array}{l}t(15) = 12.83\\ \varvec{p < .001}\\ d = 3.21\\ 95\%CI [1.48, 2.06]\end{array}$$	$$\begin{array}{l}t(15) = 8.72\\ \varvec{p < .001}\\ d = 2.18\\ 95\%CI [0.95, 1.57]\end{array}$$	$$\begin{array}{l}t(15) = -14.68\\ \varvec{p < .001}\\ d = -3.67\\ 95\%CI [-1.59, -1.19]\end{array}$$

		$$\begin{array}{l}fNIRS \Delta [HbO]\\ LABELED\end{array}$$	$$\begin{array}{l}fNIRS \Delta [HbR]\\ LABELED\end{array}$$		$$\begin{array}{l}fNIRS \Delta [HbO]\\ LABELED\end{array}$$	$$\begin{array}{l}fNIRS \Delta [HbR]\\ LABELED\end{array}$$
(mean ± SEM)		($0.62\pm 0.13$)	($-1.10\pm 0.12$)		($1.08\pm 0.17$)	($-1.39\pm 0.10$)
t-test		$$\begin{array}{l}t(15) = 4.65\\ \varvec{p < .001}\\ d = 1.16\\ 95\%CI [0.34, 0.91]\end{array}$$	$$\begin{array}{l}t(15) = -9.46\\ \varvec{p < .001}\\ d = -2.36\\ 95\%CI [-1.35, -0.85]\end{array}$$		$$\begin{array}{l}t(15) = 6.31\\ \varvec{p < .001}\\ d =1.58\\ 95\%CI [0.72, 1.45]\end{array}$$	$$\begin{array}{l}t(17) = -14.34\\ \varvec{p < .001}\\ d = -3.59\\ 95\%CI [-1.60, -1.18]\end{array}$$

fMRI PEAK $\sim$	ME RIGHT					
	M1 LEFT			M1 RIGHT		
	fMRI CHANLOCS	fNIRS $\Delta [HbO]$	fNIRS $\Delta [HbR]$	fMRI CHANLOCS	fNIRS $\Delta [HbO]$	fNIRS $\Delta [HbR]$
(mean ± SEM)	($1.76\pm 0.12$)	($1.20\pm 0.09$)	($-1.35\pm 0.11$)	($1.23\pm 0.17$)	($0.65\pm 0.17$)	($-0.89\pm 0.12$)
t-test	$$\begin{array}{l}t(15) = 14.54\\ \varvec{p < .001}\\ d = 3.63\\ 95\%CI [1.50, 2.02]\end{array}$$	$$\begin{array}{l}t(15) = 13.70\\ \varvec{p < .001}\\ d = 3.42\\ 95\%CI [1.01, 1.39]\end{array}$$	$$\begin{array}{l}t(15) = -12.18\\ \varvec{p < .001}\\ d = -3.05\\ 95\%CI [-1.59, -1.11]\end{array}$$	$$\begin{array}{l}t(15) = 7.07\\ \varvec{p < .001}\\ d = 1.77\\ 95\%CI [0.86, 1.61]\end{array}$$	$$\begin{array}{l}t(15) = 3.82\\ \varvec{p < .01}\\ d = 0.95\\ 95\%CI [0.29, 1.01]\end{array}$$	$$\begin{array}{l}t(15) = -7.19\\ \varvec{p < .001}\\ d = -1.80\\ 95\%CI [-1.16, -0.63]\end{array}$$

		$$\begin{array}{l}fNIRS \Delta [HbO]\\ LABELED\end{array}$$	$$\begin{array}{l}fNIRS \Delta [HbR]\\ LABELED\end{array}$$		$$\begin{array}{l}fNIRS \Delta [HbO]\\ LABELED\end{array}$$	$$\begin{array}{l}fNIRS \Delta [HbR]\\ LABELED\end{array}$$
(mean ± SEM)		($0.98\pm 0.14$)	($-1.33\pm 0.10$)		($0.57\pm 0.17$)	($-0.65\pm 0.17$)
t-test		$$\begin{array}{l}t(15) = 6.93\\ \varvec{p < .001}\\ d = 1.73\\ 95\%CI [0.68, 1.28]\end{array}$$	$$\begin{array}{l}t(15) = -12.79\\ \varvec{p < .001}\\ d = -3.20\\ 95\%CI [-1.55, -1.10]\end{array}$$		$$\begin{array}{l}t(15) = 3.27\\ \varvec{p < .01}\\ d = 0.82\\ 95\%CI [0.20, 0.93]\end{array}$$	$$\begin{array}{l}t(15) = -3.96\\ \varvec{p < .01}\\ d = -0.99\\ 95\%CI [-1.01, -0.30]\end{array}$$

Table contains the results of all one sample t-tests of the Fisher’s z-transformed Spearman correlation coefficients from the correlation between the individual fMRI PEAK grand average time series data with the grand average time series data of all other data types. (mean ± SEM) represents the averaged z-transformed Spearman correlation coefficients and its standard error of the mean. P-values are corrected for multiple comparisons.

**Table 2:** Within subjects: time series correlation (all tasks and SMA ROI).

fMRI PEAK $\sim$	ME LEFT			ME RIGHT		
	fMRI CHANLOCS	fNIRS $\Delta [HbO]$	fNIRS $\Delta [HbR]$	fMRI CHANLOCS	fNIRS $\Delta [HbO]$	fNIRS $\Delta [HbR]$
(mean ± SEM)	($1.02\pm 0.15$)	($0.87\pm 0.10$)	($-0.90\pm 0.10$)	($1.29\pm 0.13$)	($0.68\pm 0.18$)	($-1.08\pm 0.10$)
t-test	$$\begin{array}{l}t(15) = 6.65\\ \varvec{p < .001}\\ d = 1.66\\ 95\%CI [0.69, 1.35]\end{array}$$	$$\begin{array}{l}t(15) = 8.71\\ \varvec{p < .001}\\ d = 2.18\\ 95\%CI [0.65, 1.08]\end{array}$$	$$\begin{array}{l}t(15) = -9.04\\ \varvec{p < .001}\\ d = -2.26\\ 95\%CI [-1.11, -0.69]\end{array}$$	$$\begin{array}{l}t(15) = 9.77\\ \varvec{p < .001}\\ d = 2.44\\ 95\%CI [1.01, 1.57]\end{array}$$	$$\begin{array}{l}t(15) = 3.84\\ \varvec{p < .01}\\ d = 0.96\\ 95\%CI [0.30, 1.06]\end{array}$$	$$\begin{array}{l}t(15) = -10.84\\ \varvec{p < .001}\\ d = -2.71\\ 95\%CI [-1.29, -0.86]\end{array}$$

		$$\begin{array}{l}fNIRS \Delta [HbO]\\ LABELED\end{array}$$	$$\begin{array}{l}fNIRS \Delta [HbR]\\ LABELED\end{array}$$		$$\begin{array}{l}fNIRS \Delta [HbO]\\ LABELED\end{array}$$	$$\begin{array}{l}fNIRS \Delta [HbR]\\ LABELED\end{array}$$
(mean ± SEM)		($0.70\pm 0.13$)	($-0.85\pm 0.16$)		($0.68\pm 0.18$)	($-1.05\pm 0.12$)
t-test		$$\begin{array}{l}t(15) = 5.42\\ \varvec{p < .001}\\ d = 1.35\\ 95\%CI [0.43, 0.98]\end{array}$$	$$\begin{array}{l}t(15) = -5.30\\ \varvec{p < .001}\\ d = -1.33\\ 95\%CI [-1.20, -0.51]\end{array}$$		$$\begin{array}{l}t(15) = 3.74\\ \varvec{p < .01}\\ d = 0.94\\ 95\%CI [0.29, 1.07]\end{array}$$	$$\begin{array}{l}t(15) = -9.07\\ \varvec{p < .001}\\ d = -2.27\\ 95\%CI [-1.30, -0.80]\end{array}$$

fMRI PEAK $\sim$	MI LEFT			MI RIGHT		
	fMRI CHANLOCS	fNIRS $\Delta [HbO]$	fNIRS $\Delta [HbR]$	fMRI CHANLOCS	fNIRS $\Delta [HbO]$	fNIRS $\Delta [HbR]$
(mean ± SEM)	($0.98\pm 0.10$)	($0.57\pm 0.12$)	($-0.63\pm 0.12$)	($1.03\pm 0.16$)	($0.39\pm 0.15$)	($-0.47\pm 0.12$)
t-test	$$\begin{array}{l}t(15) = 9.90\\ \varvec{p < .001}\\ d = 2.48\\ 95\%CI [0.77, 1.19]\end{array}$$	$$\begin{array}{l}t(15) = 4.55\\ \varvec{p < .001}\\ d = 1.14\\ 95\%CI [0.30, 0.83]\end{array}$$	$$\begin{array}{l}t(15) = -5.31\\ \varvec{p < .001}\\ d = -1.33\\ 95\%CI [-0.89, -0.38]\end{array}$$	$$\begin{array}{l}t(15) = 6.60\\ \varvec{p < .001}\\ d = 1.65\\ 95\%CI [0.70, 1.36]\end{array}$$	$$\begin{array}{l}t(15) = 2.56\\ \varvec{p < .05}\\ d = 0.64\\ 95\%CI [0.07, 0.71]\end{array}$$	$$\begin{array}{l}t(15) = -4.06\\ \varvec{p < .01}\\ d = -1.01\\ 95\%CI [-0.72, -0.22]\end{array}$$

		$$\begin{array}{l}fNIRS \Delta [HbO]\\ LABELED\end{array}$$	$$\begin{array}{l}fNIRS \Delta [HbR]\\ LABELED\end{array}$$		$$\begin{array}{l}fNIRS \Delta [HbO]\\ LABELED\end{array}$$	$$\begin{array}{l}fNIRS \Delta [HbR]\\ LABELED\end{array}$$
(mean ± SEM)		($0.53\pm 0.18$)	($-0.44\pm 0.15$)		($0.33\pm 0.14$)	($-0.49\pm 0.14$)
t-test		$$\begin{array}{l}t(15) = 2.89\\ \varvec{p < .05}\\ d = 0.72\\ 95\%CI [0.14, 0.93]\end{array}$$	$$\begin{array}{l}t(15) = -3.01\\ \varvec{p < .05}\\ d = -0.75\\ 95\%CI [-0.76, -0.13]\end{array}$$		$$\begin{array}{l}t(15) = 2.44\\ \varvec{p < .05}\\ d = 0.61\\ 95\%CI [0.04, 0.62]\end{array}$$	$$\begin{array}{l}t(15) = -3.50\\ \varvec{p < .01}\\ d = -0.88\\ 95\%CI [-0.79, -0.19]\end{array}$$

	MI WHOLE BODY					
fMRI PEAK $\sim$	fMRI CHANLOCS	fNIRS $\Delta [HbO]$	fNIRS $\Delta [HbR]$			
(mean ± SEM)	($0.74\pm 0.16$)	($0.25\pm 0.14$)	($-0.74\pm 0.14$)			
t-test	$$\begin{array}{l}t(15) = 4.76\\ \varvec{p < .001}\\ d = 1.19\\ 95\%CI [0.41, 1.07]\end{array}$$	$$\begin{array}{l}t(15) = 1.74\\ p > .05\\ d = 0.43\\ 95\%CI [-0.06, 0.55]\end{array}$$	$$\begin{array}{l}t(15) = -5.43\\ \varvec{p < .001}\\ d = -1.36\\ 95\%CI [-1.02, -0.45]\end{array}$$			

		$$\begin{array}{l}fNIRS \Delta [HbO]\\ LABELED\end{array}$$	$$\begin{array}{l}fNIRS \Delta [HbR]\\ LABELED\end{array}$$			
(mean ± SEM)		($0.19\pm 0.14$)	($-0.50\pm 0.17$)			
t-test		$$\begin{array}{l}t(15) = 1.29\\ p > .05\\ d = 0.32\\ 95\%CI [-0.12, 0.49]\end{array}$$	$$\begin{array}{l}t(15) = -2.86\\ \varvec{p < .05}\\ d = -0.71\\ 95\%CI [-0.87, -0.13]\end{array}$$			

Table contains the results of all one sample t-tests of the Fisher’s z-transformed Spearman correlation coefficients from the correlation between the individual fMRI PEAK grand average time series data with the grand average time series data of all other data types. (mean±SEM) represents the averaged z-transformed Spearman correlation coefficients and its standard error of the mean. P-values are corrected for multiple comparisons.

### Task sensitivity

#### Between subjects: task-related activation patterns

**Figure 3:**
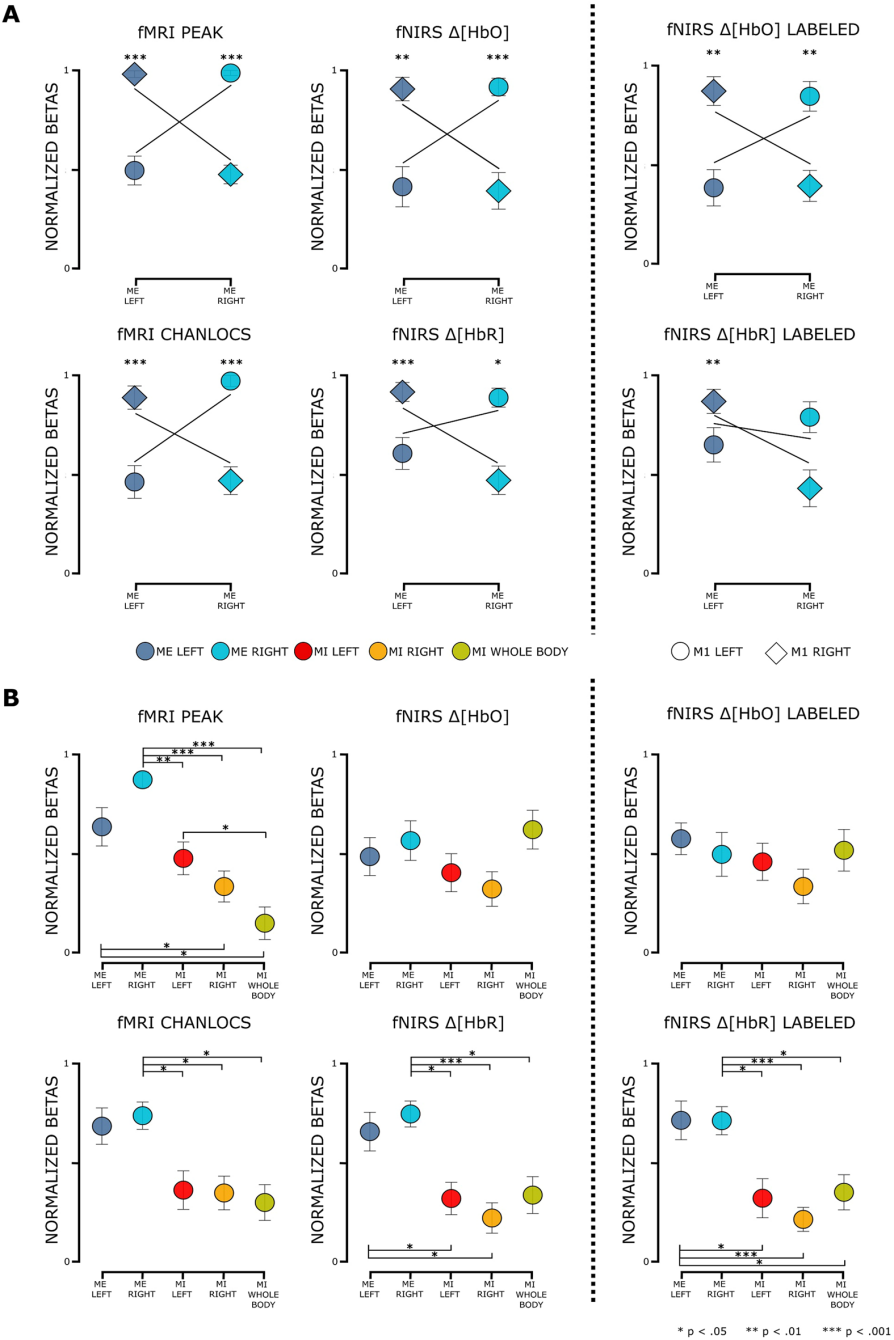
Between subjects: task related activation patterns for **(A)** ME tasks in M1 ROIs and **(B)** all tasks in ROI SMA. Illustration of mean normalized beta scores (min-max beta values) and rmANOVA results for ME LEFT (blue) and ME RIGHT (turquoise), MI LEFT (red), MI RIGHT (orange) and MI WHOLE BODY (green) tasks for fMRI PEAK, fNIRS $\Delta [HbO]$, fMRI CHANLOCS and fNIRS $\Delta [HbR]$ on the left side. On the right side of the dashed line the results from tasks fNIRS $\Delta [HbO]$ LABELED and fNIRS $\Delta [HbR]$ LABELED are shown. Note: Error bars represent standard error of the mean. Asterisks indicate significance corrected for multiple comparisons within a data type.

For ROI M1, descriptively, a clear lateralisation for both ME tasks can be seen for all data types (cf. Fig. 3A). Regarding ROI SMA (cf. Fig. 3B), all data types except for fNIRS $\Delta [HbO]$ show stronger activation in ME tasks as compared to MI tasks. The increments in activation, again descriptively, differ between data types for both ME and MI tasks.

For M1 ROIs highly significant interactions between task and hemisphere ($p < .001$) were found for all data types. Post-hoc tests comparing activation of both ME tasks within ROI M1 RIGHT were highly significant (all $p < .001$; cf. Table 3) for all data types. For ROI M1 LEFT results indicated highly significant differences between ME tasks ($p < .05$) for all data types. For detailed results see Table 3.

Except for data type fNIRS $\Delta [HbO]$, all rmANOVAs for the ROI SMA yielded a main effect of the factor task (cf. Table 4). Post-hoc tests revealed mixed results for pair-wise comparisons (cf. Fig. 3B and Table 4). However, no pair-wise comparison indicated significant differences between ME LEFT and ME RIGHT tasks. With respect to the MI tasks, only data type fMRI PEAK revealed a significant difference between MI LEFT and MI WHOLE BODY ($t(15) = 2.99$, $p < .05$, $d = 0.75$). For fNIRS data types, only for $\Delta [HbR]$ ME task activation was significantly stronger than MI task activation.

##### Channel labeling

With respect to ME tasks in M1, after adding anatomical information the comparison between ME LEFT and ME RIGHT in ROI M1 LEFT of fNIRS $\Delta [HbR]$ LABELED was not significant anymore, however, all other comparisons were similar to the unlabeled versions (cf. Table 3). For ROI SMA, results of both $\Delta [HbR]$ and $\Delta [HbO]$ LABELED were comparable to those of the unlabeled data types (cf. Table 4).

**Table 3:** Between subjects: task-related activation patterns (ME tasks and M1 ROIs).

	fMRI PEAK	$$\begin{array}{l}\hbox {M1 LEFT}\\ \hbox {ME LEFT} \sim \hbox {ME RIGHT}\end{array}$$		fMRI CHANLOCS	$$\begin{array}{l}\hbox {M1 LEFT}\\ \hbox {ME LEFT} \sim \hbox {ME RIGHT}\end{array}$$	
Task	$$\begin{array}{l}F(1, 15) = 0.03\\ p > .05\\ \eta _{p}^{2} = 0.00\end{array}$$	(mean ± SEM)	$$\begin{array}{l}\hbox {ME LEFT}: (0.50\pm 0.07)\\ \hbox {ME RIGHT}: (0.99\pm 0.01)\end{array}$$	$$\begin{array}{l}F(1, 15) = 0.41\\ p > .05\\ \eta _{p}^{2} = 0.03\end{array}$$	(mean ± SEM)	(mean±SEM) $$\begin{array}{l}\hbox {ME LEFT}: (0.47\pm 0.07)\\ \hbox {ME RIGHT}: (0.97\pm 0.03)\end{array}$$
Hemisphere	$$\begin{array}{l}F(1, 15) = 0.10\\ p > .05\\ \eta _{p}^{2} = 0.00\end{array}$$	t-test	$$\begin{array}{l}t(15) = -6.62\\ \varvec{p < .001}\\d = -1.67\end{array}$$	$$\begin{array}{l}F(1, 15) = 0.33\\ p > .05\\ \eta _{p}^{2} = 0.02\end{array}$$	t-test	$$\begin{array}{l}t(15) = -5.81\\ \varvec{p < .001}\\d = -1.45\end{array}$$
$$\begin{array}{l}\mathrm{Task:}\\ \mathrm{Hemisphere}\end{array}$$	$$\begin{array}{l}F(1, 15) = 102.81\\ \varvec{p < .001}\\ \eta _{p}^{2} = 0.87\end{array}$$	$$\begin{array}{l}\hbox {M1 RIGHT}\\ \hbox {ME LEFT} \sim \hbox {ME RIGHT}\end{array}$$		$$\begin{array}{l}F(1, 15) = 71.37\\ \varvec{p < .001}\\ \eta _{p}^{2} = 0.83\end{array}$$	$$\begin{array}{l}\hbox {M1 RIGHT}\\ \hbox {ME LEFT} \sim \hbox {ME RIGHT}\end{array}$$	
		(mean ± SEM)	$$\begin{array}{l}\hbox {ME LEFT}: (0.98\pm 0.02)\\ \hbox {ME RIGHT}: (0.48\pm 0.05)\end{array}$$		(mean ± SEM)	$$\begin{array}{l}\hbox {ME LEFT}: (0.89\pm 0.06)\\ \hbox {ME RIGHT}: (0.47\pm 0.07)\end{array}$$
		t-test	$$\begin{array}{l}{t(15)} = 9.48\\ \varvec{p < .001}\\ d = 2.37\end{array}$$		t-test	$$\begin{array}{l}t(15) = 4.59\\ \varvec{p < .001}\\ d = 1.15\end{array}$$
	fNIRS $\Delta [HbO]$	$$\begin{array}{l}\hbox {M1 LEFT}\\ \hbox {ME LEFT} \sim \hbox {ME RIGHT}\end{array}$$		fNIRS $\Delta [HbR]$	$$\begin{array}{l}\hbox {M1 LEFT}\\ \hbox {ME LEFT} \sim \hbox {ME RIGHT}\end{array}$$	
Task	$$\begin{array}{l}F(1, 15) = 0.00\\ p > .05\\ \eta _{p}^{2} = 0.00\end{array}$$	(mean ± SEM)	$$\begin{array}{l}\hbox {ME LEFT}: (0.42 \pm 0.10)\\ \hbox {ME RIGHT}: (0.92 \pm 0.04)\end{array}$$	$$\begin{array}{l}F(1, 15) = 1.03\\ p > .05\\ \eta _{p}^{2} = 0.06\end{array}$$	(mean ± SEM)	$$\begin{array}{l}\hbox {ME LEFT}: (0.61 \pm 0 .08)\\ \hbox {ME RIGHT:} (0.89 \pm 0.05)\end{array}$$
Hemisphere	$$\begin{array}{l}F(1, 15) = 0.06\\ p > .05\\ \eta _{p}^{2} = 0.00\end{array}$$	t-test	$$\begin{array}{l}t(15) = - 4.96 \\ \varvec{p < .001}\\{\text{d = }} - {\text{1.24}} \\ \end{array}$$	$$\begin{array}{l}F(1, 15) = 1.37\\ p > .05\\ \eta _{p}^{2} = 0.08\end{array}$$	t-test	$$\begin{array}{l}t(15) = -2.45\\ \varvec{p < .05}\\ d = -0.61\end{array}$$
$$\begin{array}{l}\mathrm{Task:}\\ \mathrm{Hemisphere}\end{array}$$	$$\begin{array}{l}F(1, 15) = 45.47\\ \varvec{p < .001}\\ \eta _{p}^{2} = 0.75\end{array}$$	$$\begin{array}{l}\hbox {M1 RIGHT}\\ \hbox {ME LEFT} \sim \hbox {ME RIGHT}\end{array}$$		$$\begin{array}{l}F(1, 15) = 33.62\\ \varvec{p < .001}\\ \eta _{p}^{2} = 0.69\end{array}$$	$$\begin{array}{l}\hbox {M1 RIGHT}\\ \hbox {ME LEFT} \sim \hbox {ME RIGHT}\end{array}$$	
		(mean ± SEM)	$$\begin{array}{l}\hbox {ME LEFT}: (0.91\pm 0.06)\\ \hbox {ME RIGHT}: (0.40\pm 0.09)\end{array}$$		(mean ± SEM)	$$\begin{array}{l}\hbox {ME LEFT}: (0.92\pm 0.05)\\ \hbox {ME RIGHT}: (0.47\pm 0.07)\end{array}$$
		t-test	$$\begin{array}{l}t(15) = 3.79\\ \varvec{p < .01}\\d = 0.95\end{array}$$		t-test	$$\begin{array}{l}t(15) = 5.09\\ \varvec{p <.001}\\d = 1.27\end{array}$$
	fNIRS $\Delta [HbO]$ LABELED	$$\begin{array}{l}\hbox {M1 LEFT}\\ \hbox {ME LEFT} \sim \hbox {ME RIGHT}\end{array}$$		fNIRS $\Delta [HbR]$ LABELED	$$\begin{array}{l}\hbox {M1 LEFT}\\ \hbox {ME LEFT} \sim \hbox {ME RIGHT}\end{array}$$	
Task	$$\begin{array}{l}F(1, 15) = 0.01\\ p > .05\\ \eta _{p}^{2} = 0.00\end{array}$$	(mean ± SEM)	$$\begin{array}{l}\hbox {ME LEFT}: (0.39 \pm 0.09)\\ \hbox {ME RIGHT}: (0.85 \pm 0.07)\end{array}$$	$$\begin{array}{l}F(1, 15) = 2.20\\ p > .05\\ \eta _{p}^{2} = 0.13\end{array}$$	(mean ± SEM)	$$\begin{array}{l}\hbox {ME LEFT}: (0.65 \pm 0.09)\\ \hbox {ME RIGHT}: (0.79 \pm 0.08)\end{array}$$
Hemisphere	$$\begin{array}{l}F(1, 15) = 0.07\\ p > .05\\ \eta _{p}^{2} = 0.00\end{array}$$	t-test	$$\begin{array}{l}t(15) = -3.97\\ \varvec{p < .01}\\d = -0.99\end{array}$$	$$\begin{array}{l}F(1, 15) = 1.38\\ p > .05\\ \eta _{p}^{2} = 0.08\end{array}$$	t-test	$$\begin{array}{l}t(15) = -1.03\\p > .05\\d = -0.26\end{array}$$
$$\begin{array}{l}\mathrm{Task:}\\ \mathrm{Hemisphere}\end{array}$$	$$\begin{array}{l}F(1, 15) = 50.61\\ \varvec{p < .001}\\ \eta _{p}^{2} = 0.77\end{array}$$	$$\begin{array}{l}\hbox {M1 RIGHT}\\ \hbox {ME LEFT} \sim \hbox {ME RIGHT}\end{array}$$		$$\begin{array}{l}F(1, 15) = 14.94\\ \varvec{p < .01}\\ \eta _{p}^{2} = 0.50\end{array}$$	$$\begin{array}{l}\hbox {M1 RIGHT}\\ \hbox {ME LEFT} \sim \hbox {ME RIGHT}\end{array}$$	
		(mean ± SEM)	$$\begin{array}{l}\hbox {ME LEFT}: (0.87 \pm 0.07)\\ \hbox {ME RIGHT}: (0.40 \pm 0.08)\end{array}$$		(mean ± SEM)	$$\begin{array}{l}\hbox {ME LEFT}: (0.87 \pm 0.06)\\ \hbox {ME RIGHT}: (0.43 \pm 0.09)\end{array}$$
		t-test	$$\begin{array}{l}t(15) = 3.86\\ \varvec{p <.01}\\d = 0.97\end{array}$$		t-test	$$\begin{array}{l}t(15) = 3.81\\ \varvec{p < .01}\\d = 0.95\end{array}$$

Table contains the results of all $2\times 2$ repeated measures ANOVAs with min–max normalized betas of both ME tasks as dependent variables. (mean±SEM) represents the averaged min–max normalised beta values and its standard error of the mean. P-values are corrected for multiple comparisons.

**Table 4:** Between subjects: task-related activation patterns (ME and MI tasks and ROI SMA).

Task	fMRI PEAK					fMRI CHANLOCS				
	$F(2.95, 44.17) = 11.12$, $\varvec{p < .001}$, $\eta _{p}^{2} = 0.43$					$F(3.20, 48.04) = 4.94$, $\varvec{p < .01}$, $\eta _{p}^{2} = 0.25$				
	**Post hoc tests**					**Post hoc tests**				
	$$\begin{array}{l}\hbox {ME LEFT}\\ (0.64 \pm 0.10)\end{array}$$	$$\begin{array}{l}\hbox {ME RIGHT}\\ (0.87 \pm 0.04)\end{array}$$	$$\begin{array}{l}\hbox {MI LEFT}\\ (0.48 \pm 0.08)\end{array}$$	$$\begin{array}{l}\hbox {MI RIGHT}\\ (0.34 \pm 0.08)\end{array}$$	$$\begin{array}{l}\hbox {MI WHOLE BODY}\\ (0.15 \pm 0.08)\end{array}$$	$$\begin{array}{l}\hbox {ME LEFT}\\ (0.69 \pm 0.09)\end{array}$$	$$\begin{array}{l}\hbox {ME RIGHT}\\ (0.74 \pm 0.07)\end{array}$$	$$\begin{array}{l}\hbox {MI LEFT}\\ (0.36 \pm 0.10)\end{array}$$	$$\begin{array}{l}\hbox {MI RIGHT}\\ (0.35 \pm 0.09)\end{array}$$	$$\begin{array}{l}\hbox {MI WHOLE BODY}\\ (0.30 \pm 0.09)\end{array}$$
ME LEFT	−	$$\begin{array}{l}t(15) = -2.30\\ p > .05\\ d = -0.57\end{array}$$	$$\begin{array}{l}t(15) = 1.10\\ p > .05\\ d = 0.27\end{array}$$	$$\begin{array}{l}t(15) = 2.52\\ \varvec{p < .05}\\ d = 0.63\end{array}$$	$$\begin{array}{l}t(15) = 3.16\\ \varvec{p < .05}\\ d = 0.79\end{array}$$	−	$$\begin{array}{l}t(15) = -0.42\\ p > .05\\ d = -0.10\end{array}$$	$$\begin{array}{l}t(15) = 1.99\\ p > .05\\ d = 0.50\end{array}$$	$$\begin{array}{l}t(15) = 2.29\\ p > .05\\ d = 0.57\end{array}$$	$$\begin{array}{l}t(15) = 2.37\\ p > .05\\ d = 0.59\end{array}$$
ME RIGHT	−	−	$$\begin{array}{l}t(15) = 3.71\\ \varvec{p < .01}\\ d = 0.93\end{array}$$	$$\begin{array}{l}t(15) = 6.17\\ \varvec{p < .001}\\ d = 1.54\end{array}$$	$$\begin{array}{l}t(15) = 7.32\\ \varvec{p < .001}\\ d = 1.83\end{array}$$	−	−	$$\begin{array}{l}t(15) = 2.87\\ \varvec{p < .05}\\ d = 0.72\end{array}$$	$$\begin{array}{l}t(15) = 3.53\\ \varvec{p < .05}\\ d = 0.88\end{array}$$	$$\begin{array}{l}t(15) = 3.97\\ \varvec{p < .05}\\ d = 0.99\end{array}$$
MI LEFT	−	−	−	$$\begin{array}{l}t(15) = 1.37\\ p > .05\\ d = 0.34\end{array}$$	$$\begin{array}{l}t(15) = 2.99\\ \varvec{p < .05}\\ d = 0.75\end{array}$$	−	−	−	$$\begin{array}{l}t(15) = 0.14\\ p > .05\\ d = 0.03\end{array}$$	$$\begin{array}{l}t(15) = 0.50\\ p > .05\\ d = 0.13\end{array}$$
MI RIGHT	–	–	–	–	$$\begin{array}{l}t(15) = 1.41\\ p > .05\\ d = 0.35\end{array}$$	−	−	−	−	$$\begin{array}{l}t(15) = 0.39\\ p > .05\\ d = 0.10\end{array}$$

Task	fNIRS $\Delta [HbO]$					fNIRS $\Delta [HbR]$				
	$F(3.38, 50.62) = 1.42$, $p > .05$, $\eta _{p}^{2} = 0.09$					$F(3.67, 55.04) = 6.79$, $\varvec{p < .001}$, $\eta _{p}^{2} = 0.31$				
	**Post hoc tests**					**Post hoc tests**				
	$$\begin{array}{l}\hbox {ME LEFT}\\ (0.49 \pm 0.10)\end{array}$$	$$\begin{array}{l}\hbox {ME RIGHT}\\ (0.57 \pm 0.10)\end{array}$$	$$\begin{array}{l}\hbox {MI LEFT}\\ (0.41 \pm 0.10)\end{array}$$	$$\begin{array}{l}\hbox {MI RIGHT}\\ (0.32 \pm 0.09)\end{array}$$	$$\begin{array}{l}\hbox {MI WHOLE BODY}\\ (0.62 \pm 0.10)\end{array}$$	$$\begin{array}{l}\hbox {ME LEFT}\\ (0.66 \pm 0.10)\end{array}$$	$$\begin{array}{l}\hbox {ME RIGHT}\\ (0.75 \pm 0.07)\end{array}$$	$$\begin{array}{l}\hbox {MI LEFT}\\ (0.32 \pm 0.08)\end{array}$$	$$\begin{array}{l}\hbox {MI RIGHT}\\ (0.22 \pm 0.08)\end{array}$$	$$\begin{array}{l}\hbox {MI WHOLE BODY}\\ (0.34 \pm 0.09)\end{array}$$
ME LEFT	−	−	−	−	−	−	$$\begin{array}{l}t(15) = -0.69\\ p > .05\\ d = -0.17\end{array}$$	$$\begin{array}{l}t(15) = 2.62\\ \varvec{p < .05}\\ d = 0.66\end{array}$$	$$\begin{array}{l}t(15) = 3.40\\ \varvec{p < .05}\\ d = 0.85\end{array}$$	$$\begin{array}{l}t(15) = 2.25\\ p > .05\\ d = 0.56\end{array}$$
ME RIGHT	−	−	−	−	−	−	−	$$\begin{array}{l}t(15) = 3.47\\ \varvec{p < .05}\\ d = 0.87\end{array}$$	$$\begin{array}{l}t(15) = 5.71\\ \varvec{p < .001}\\ d = 1.43\end{array}$$	$$\begin{array}{l}t(15) = 3.28\\ \varvec{p < .05}\\ d = 0.82\end{array}$$
MI LEFT	–	–	–	–	–	–	–	–	$$\begin{array}{l}t(15) = 0.82\\ p > .05\\ d = 0.21\end{array}$$	$$\begin{array}{l}t(15) = -0.13\\ p > .05\\ d = -0.03\end{array}$$
MI RIGHT	–	–	–	–	–	–	–	–	–	$$\begin{array}{l}t(15) = -0.97\\ p > .05\\ d = -0.24\end{array}$$

Task	fNIRS $\Delta [HbO]$ LABELED					fNIRS $\Delta [HbR]$ LABELED				
	$F(2.87, 43.01) = 0.75$, $p > .05$, $\eta _{p}^{2} = 0.05$					$F(3.26, 48.94) = 6.95$, $\varvec{p < .001}$, $\eta _{p}^{2} = 0.32$				
	**Post hoc tests**					**Post hoc tests**				
	$$\begin{array}{l}\hbox {ME LEFT}\\ (0.58 \pm 0.08)\end{array}$$	$$\begin{array}{l}\hbox {ME RIGHT}\\ (0.50 \pm 0.11)\end{array}$$	$$\begin{array}{l}\hbox {MI LEFT}\\ (0.46 \pm 0.09)\end{array}$$	$$\begin{array}{l}\hbox {MI RIGHT}\\ (0.34 \pm 0.09)\end{array}$$	$$\begin{array}{l}\hbox {MI WHOLE BODY}\\ (0.52 \pm 0.11)\end{array}$$	$$\begin{array}{l}\hbox {ME LEFT}\\ (0.72 \pm 0.10)\end{array}$$	$$\begin{array}{l}\hbox {ME RIGHT}\\ (0.71 \pm 0.07)\end{array}$$	$$\begin{array}{l}\hbox {MI LEFT}\\ (0.32 \pm 0.10)\end{array}$$	$$\begin{array}{l}\hbox {MI RIGHT}\\ (0.22 \pm 0.06)\end{array}$$	$$\begin{array}{l}\hbox {MI WHOLE BODY}\\ (0.35 \pm 0.09)\end{array}$$
ME LEFT	−	−	−	−	−	−	$$\begin{array}{l}t(15) = 0.01\\ p > .05\\ d = 0.00\end{array}$$	$$\begin{array}{l}t(15) = 2.75\\ \varvec{p < .05}\\ d = 0.69\end{array}$$	$$\begin{array}{l}t(15) = 5.04\\ \varvec{p < .001}\\ d = 1.26\end{array}$$	$$\begin{array}{l}t(15) = 2.61\\ \varvec{p < .05}\\ d = 0.65\end{array}$$
ME RIGHT	−	−	−	−	−	−	−	$$\begin{array}{l}t(15) = 2.66\\ \varvec{p < .05}\\ d = 0.66\end{array}$$	$$\begin{array}{l}t(15) = 5.79\\ \varvec{p < .001}\\ d = 1.45\end{array}$$	$$\begin{array}{l}t(15) = 2.97\\ \varvec{p < .05}\\ d = 0.74\end{array}$$
MI LEFT	–	–	–	–	–	–	–	−	$$\begin{array}{l}t(15) = 0.82\\ p > .05\\ d = 0.21\end{array}$$	$$\begin{array}{l}t(15) = -0.24\\ p > .05\\ d = -0.06\end{array}$$
MI RIGHT	–	–	–	–	–	–	–	–	–	$$\begin{array}{l}t(15) = -1.15\\ p > .05\\ d = -0.29\end{array}$$

Table contains the results of all repeated measures ANOVAs with min-max normalized betas as dependent variables. (mean±SEM) represents the averaged min–max normalised beta values and its standard error of the mean. P-values are corrected for multiple comparisons.

#### Within subjects: repeated measures correlation

Within subjects task sensitivity was explored by means of repeated measures correlation^33^ analyses for each ROI. Correlations were conducted between the fMRI PEAK data type and all other data types.

For ROIs M1 LEFT and M1 RIGHT the strongest positive relationship of normalized beta values was observed between fMRI PEAK and fMRI CHANLOCS (M1 LEFT: $r_{(63)} = 0.89$, $95\%$ CI [0.83, 0.93], $p < .001$; M1 RIGHT: $r_{(63)} = 0.88$, $95\%$ CI [0.80, 0.92], $p < .001$). Also, fNIRS $\Delta [HbO]$ (M1 LEFT: $r_{(63)} = 0.47$, $95\%$ CI [0.25, 0.64], $p < .001$; M1 RIGHT: $r_{(63)} = 0.66$, $95\%$ CI [0.49, 0.78], $p < .001$) and fNIRS $\Delta [HbR]$ (M1 LEFT: $r_{(63)} = 0.62$, $95\%$ CI [0.45, 0.76], $p < .001$; M1 RIGHT:$r_{(63)} = 0.66$, $95\%$ CI [0.50, 0.78], $p < .001$) showed strong positive relationships with the fMRI PEAK beta values.

Concerning ROI SMA, likewise, the strongest positive relationship between beta values was observed for fMRI PEAK and fMRI CHANLOCS data types($r_{(63)} = 0.71$, $95\%$ CI [0.55, 0.81], $p < .001$). For fNIRS $\Delta [HbO]$ ($r_{(63)} = 0.06$, $95\%$ CI $[-0.19, 0.30]$, $p > .05$) and fNIRS $\Delta [HbR]$ ($r_{(63)} = 0.29$, $95\%$ CI [0.05, 0.50], $p < .05$) repeated measures correlation coefficients were highly reduced and only significant for fNIRS $\Delta [HbR]$.

##### Channel labeling

 Overall, in M1 and SMA the results of the repeated measures correlations between fMRI PEAK and fNIRS LABELED were comparable to the unlabeled versions (fNIRS $\Delta [HbO]$ LABELED M1 LEFT: $r_{(63)} = 0.41$, $95\%$ CI [0.18, 0.60], $p < .001$, M1 RIGHT: $r_{(63)} = 0.55$, $95\%$ CI [0.35, 0.70], $p < .001$, SMA: $r_{(63)} = 0.16$, $95\%$ CI $[-0.09, 0.39]$, $p > .05$; fNIRS $\Delta [HbR]$ LABELED M1 LEFT: $r_{(63)} = 0.53$, $95\%$ CI [0.32, 0.69], $p < .001$, M1 RIGHT: $r_{(63)} = 0.56$, $95\%$ CI [0.37, 0.71], $p < .001$, SMA: $r_{(63)} = 0.33$, $95\%$ CI [0.09, 0.53], $p < .05$) .

## Discussion

The present study aimed at validating CW-fNIRS SMA recordings for ME and MI. We expected that fNIRS data would show good spatial specificity and task sensitivity, thereby matching fMRI data, which served as a basis for comparison.

### Validation of the general procedure: M1 lateralisation

Motor execution in general and finger tapping tasks in particular are well established and typically show a stronger activation in the hemisphere contralateral to the performing hand as compared to the ipsilateral hemisphere^34–36^. In the present study, both $\Delta [HbO]$ and $\Delta [HbR]$ signal types showed the expected lateralisation pattern at the group level. Moreover, time series correlations and repeated measures correlations confirmed a very good match between fMRI and fNIRS data within subjects. These results confirm spatial specificity and task sensitivity for fNIRS measurements of primary motor areas during motor execution.

### Validation of SMA activation

At the between-subjects level spatial specificity was determined by comparing fMRI CHANLOCS beta maps with the beta maps of $\Delta [HbO]$ and $\Delta [HbR]$. Maps covered SMA and left and right M1. Descriptively and in most cases statistically, analyses indicated a very good spatial match for ME task maps. For MI task maps the match was reduced. Here, only the spatial correlations of MI RIGHT reached significance. Notably, at a descriptive level, fNIRS SMA activation was most specific across data types for the MI WHOLE BODY task. Within-subjects time-series correlations for the SMA indicated that for ME tasks, correlations were comparably high for fMRI CHANLOCS on the one hand, and fNIRS data types on the other, indicating an excellent match between fMRI PEAK and channel data. With respect to the MI task correlations, again, all time-series correlations were significant with the one exception of $\Delta [HbO]$ in the MI WHOLE BODY task.

Within-subjects analyses of task sensitivity indicated that the task activation pattern of fMRI PEAK was, unsurprisingly, most closely matched by fMRI CHANLOCS data. Task sensitivity was reduced for both fNIRS data types. Across all data types between-subjects analyses showed that ME was generally associated with stronger activation than MI. However, pairwise comparisons indicated that even for fMRI PEAK data, this basic difference was not significant for all ME-MI pairs. Within MI tasks, fMRI PEAK data suggested a stepwise reduction in activation from MI LEFT to MI RIGHT to MI WHOLE BODY. Notably, this reduction was only significant between tasks MI LEFT and MI WHOLE BODY. For fMRI CHANLOCS activation seemed very comparable across the three MI tasks, whereas for fNIRS data types, descriptively, MI WHOLE BODY was associated with the strongest activation, followed by MI LEFT and MI RIGHT. However, none of the pairwise comparisons was significant. Thus, even though in terms of activation strength MI WHOLE BODY seems to be the winner for fNIRS, regarding MI task sensitivity the results remain inconclusive.

In summary, the results indicate that spatial specificity of fNIRS is strong for ME tasks for both $\Delta [HbO]$ and $\Delta [HbR]$. For MI tasks spatial specificity seems generally lower, although the combination of $\Delta [HbR]$ and MI WHOLE BODY revealed the highest spatial specificity on a within subjects level. Task sensitivity was stronger for $\Delta [HbR]$ as compared to $\Delta [HbO]$ at both within and between subjects level. Dravida et al.^37^ observed an overall stronger (test–retest) spatial specificity for $\Delta [HbR]$, whereas task-related (test–retest) reliability was stronger for $\Delta [HbO]$. The authors argued that the results were not only dependent on the signal type, but also on the performed (motor execution) task. Moreover, results improved after fNIRS data was corrected for systemic activity^37^, emphasizing the significance of applying adequate correction methods in order to increase spatial specificity and task sensitivity in fNIRS research. The results of the present study also indicate that differences between $\Delta [HbO]$ and $\Delta [HbR]$ in spatial specificity and task sensitivity are task- and signal-dependent. However, fNIRS and fMRI studies comparing different MI tasks are lacking, and most existing fNIRS studies that included a MI task did not report $\Delta [HbR]$, making it difficult to compare the present findings. Furthermore, there is a lack of research on the neural correlates of MI of whole body movements. To the best of our knowledge, only one fMRI study compared MI of complex (everyday) upper limb and whole body movements^38^. Although the results revealed that both MI tasks activate similar brain areas as MI of hand/finger movements including the SMA, no direct comparison with hand/finger movements was conducted, which again limits comparability with the present study.

Notably, even for ME tasks, spatial specificity and task sensitivity of fNIRS data was somewhat reduced as compared to fMRI PEAK data. This could be taken as evidence for a general shortcoming of the CW-NIRS. However, differences in activation patterns were not only present between fMRI PEAK and fNIRS data, but already between fMRI PEAK and fMRI CHANLOCS data. This indicates that to some degree, differences between fMRI PEAK and fNIRS data result from the distance between the location of a channel and that of the fMRI PEAK activation. Differences between fNIRS channel data types and fMRI CHANLOCS could indicate that the 5 mm sphere used to extract voxels for fMRI CHANLOCS included voxels that were not reached by the near infrared light, but were closer to the fMRI PEAK location. A more precise way of estimating the involved voxels for each channel could be to simulate the propagation of the near infrared light in the tissue^29,39,40^.

### Individual anatomical information: labeled channels

A limitation of fNIRS is the lack of anatomical information^4^. In order to assess how individual anatomical information affects spatial specificity and task sensitivity, fNIRS data were also analysed only for those channels for which co-registration analysis confirmed that they covered either M1 or SMA. Overall, results did not change remarkably for the labeled channel set. However, as a general tendency effect sizes tended to decrease. This effect may indicate potential inaccuracies in cap placement. That is, higher effect sizes of the unlabeled fNIRS data types may result from picking up activity from neighboring but stronger activated brain regions (e.g., a channel supposed to cover the SMA and therefore picked for SMA data analysis actually records data from M1). Of course, the same problem could arise due to individual differences in brain anatomy. In order to minimize inaccuracies resulting from cap placement, anatomical landmarks should always be incorporated when placing the cap^41^. Besides, a suitable tool can help to design an optode layout and verify correct placement with respect to the regions of interest (e.g., fOLD^5^; AtlasViewer^6^). Additionally and if available, the application of a 3D digitizer should be considered in order to control for satisfying individual cap placement (e.g., with AtlasViewer). Subjects with poor cap placement can thus be detected and excluded from further analyses.

Taken together, for the present data set, individual anatomical information did not increase spatial specificity and task sensitivity of fNIRS data in motor regions for ME and MI tasks. Whether this result is transferable to other brain regions remains to be shown.

### Which fNIRS signal should be used for neurorehabilitation purposes?

With respect to ME tasks and motor regions, several CW-fNIRS-fMRI co-registration studies set out to identify the signal type with the strongest relationship to fMRI BOLD. However, findings are ambiguous as in some cases $\Delta [HbR]$^22^ was most strongly related with fMRI BOLD, while in others it was $\Delta [HbO]$^25,27^. A possible reason for this inconsistency may be the lack of systemic activity correction in these early studies. It is well known that systemic artifacts can be distributed over the head and show complex characteristics within and between subjects as well as across tasks^3,7,12,37^. If not appropriately taken care of, the artifact is likely to affect the experimental effects^9,10^. Problems arising from systemic artifacts are assumed to be stronger for $\Delta [HbO]$ than $\Delta [HbR]$^7,37^, though weaker is not equivalent to absent^9^.

In the present study short-distance channel correction was applied in order to minimize systemic activity in the CW-fNIRS signal. When the aim is to use MI in combination with SMA neurofeedback, based on these data we suggest to give preference to $\Delta [HbR]$, in particular when used with MI of whole body movements. Also, when considering all results together, for MI of finger/hand movements $\Delta [HbR]$ might be the better choice. When ME generated signals are relevant, then the results of our analysis indicate a slightly stronger task sensitivity for $\Delta [HbO]$, but only for the M1 and not for the SMA region. However, our study does not support a firm conclusion on the question of which fNIRS signal type to use for neurorehabilitation neurofeedback. Rather, our study demonstrates the importance of critically evaluating the choice of signal in a particular fNIRS neurofeedback application.

### Limitations

Our study has several limitations. One is that due to hardware limitations fNIRS and fMRI were recorded consecutively, not concurrently. Simultaneously recorded data would not increase the spatial specificity or task sensitivity of fNIRS measurements per se, but would improve the accuracy of co-registration and be particularly useful for understanding discrepancies between fMRI and fNIRS results. Based on this lack of simultaneous measurements, it is possible that the performance of the ME and MI tasks between sessions differed. This variability in task performance between data types could have influenced the results of the present study.

In this work, an optical digitizer was used to collect individual optode and marker point positions for fNIRS recordings. The optical digitizer works with two cameras that detect the position of a pointing stick. However, the field of view of the digitizer’s camera was too narrow, for which reason the connection to the pointing stick was lost numerous times. Compensatory movements of the stick resulted in position inaccuracies. To account for these inaccuracies, a correction method based on the individual 10-5 positions generated by AtlasViewer and a rigid transformation was applied. However, this correction method introduced another potential source of error because individual anatomical locations have to be selected manually in the AtlasViewer GUI.

For the filter settings the recommendations of Pinti et al. were followed, that is, a FIR bandpass filter with cut-off frequencies of [0.01, 0.09] Hz and a filter order of 1000. The lower cut-off frequency of 0.01 Hz was chosen to not attenuate the task frequency ($0.0270-0.0303$ Hz). The filter did likely not account for very low frequency oscillations which typically overlap with or are very close to the task frequency and are therefore difficult to remove^42^.

One question raised in this study is the choice of fNIRS signal for SMA neurofeedback. We restricted our analyses to $\Delta [HbO]$ or $\Delta [HbR]$ because these are the most used signals in this area of research. Other potentially interesting signal types not taken into consideration here would be total hemoglobin concentration changes^4^ (i.e., $\Delta [HbT]$ = $\Delta [HbO]$ + $\Delta [HbR]$) or the difference between $\Delta [HbO]$ and $\Delta [HbR]$^43^ (i.e., $\Delta [HbDiff]$ = $\Delta [HbO]$ − $\Delta [HbR]$).

### Conclusions

We validated CW-fNIRS SMA recordings by comparing their spatial specificity and task sensitivity to fMRI. Spatial specificity and task sensitivity were in many aspects comparable to fMRI, though some fluctuation was evident for particular combinations of MI task and fNIRS measure. We nonetheless conclude that CW-fNIRS can be employed in SMA neurofeedback in corresponding setups. We used short-distance channels to correct for systemic artifacts in the fNIRS data. The correction improves our confidence in the present results, in particular in relation to earlier reports that did not apply any systemic artifact correction. We think it is important that future fNIRS studies with or without neurofeedback similarly employ systemic artifact correction. Ideally, this will be based on short-distance channel recordings, which we believe will quickly become standard. Because of the advance in fNIRS signal preprocessing and because of the observed fluctuations in results for particular combinations of task and fNIRS measure we conclude that fNIRS paradigms will continue to benefit from systematic validation by fMRI, in particular if they target a specific cortical region.

## Methods

### Subjects

Participants were recruited by way of advertisement in local newspapers. In one session fMRI data and in the other session fNIRS data were collected. Based on the study by Abdalmalak et al.^31^, we aimed for at least 15 data sets with both valid fNIRS and valid fMRI data. In total, 34 participants (17 females, 17 males) participated in the study. Handedness was assessed by means of the Edinburgh Handedness Inventory (EHI^44^). All participants were right handed with a mean laterality quotient of 95.50 ± 8.89 (mean ± SD; range: 71 to 100). To exclude mild cognitive decline, the Montreal Cognitive Assessement (MoCA; all subjects in the final sample > 25 points) was applied which ranges from 0 to 30 with higher scores indicating better performance. Two individuals were excluded because of a MoCa score below 25 points. EHI and MoCA were only conducted in the first session. Furthermore, the Kinesthetic and Visual Imagery Questionnaire was conducted in both sessions before start of the experiment in order to familiarize the subjects with the general concept of motor imagery and to clarify the difference between visual and kinesthetic motor imagery.

Only participants without a history of psychological, psychiatric and neurological disorders, with normal or corrected-to-normal vision and without any experience in piano playing were included. After explanation of the study, all subjects gave written informed consent. Participants were paid 10 Euros/h as reimbursement. In accordance to the Declaration of Helsinki, the study was approved by the Medical Ethics Committee of the University of Oldenburg (permit number: 2017-139).

### Experimental design

Subjects participated in one fMRI and one fNIRS session which were at least 14 days apart (mean ± SD: [17.38 ± 3.50] days; range: 14 to 25 days) in order to avoid habituation. The experimental design was identical in both sessions. The order of the sessions was pseudo-randomized, resulting in eight subjects starting with the fNIRS and eight subjects starting with the fMRI session. For both sessions, the main experiment consisted of five different tasks. Two tasks were ME tasks and three were kinesthetic MI tasks (cf. Fig. 1B in supplementary material). The main task for both ME and MI tasks was a self-paced 5-position finger tapping task (sequence: 4-1-3-2-4) with either the left or the right hand (ME LEFT, ME RIGHT and MI LEFT, MI RIGHT). In this task each finger was assigned a key on a number pad (1 $=$ index finger, 2 $=$ middle finger, 3 $=$ ring finger, 4 $=$ little finger). Keys had to be pressed in a pre-specified order (cf. Fig. 1A in supplementary material) and continuously for the duration of a task block. In a familiarization phase participants learned and practiced the finger tapping sequence with each hand until they memorized the sequence. The fifth task was MI of a self-selected bilateral whole body movement (MI WHOLE BODY). The only restriction for this task was that the imagined movement included both arms and legs (e.g., swimming) and that the subject had experience in performing the movement. The experimenter asked the participants to provide own ideas for movements and did not provide examples. The order of task runs was pseudo-randomized for each session with the restriction that not all three MI tasks were presented consecutively in order to reduce fatigue.

A task block always started with the task instruction for the task block (e.g. ‘BEWEGEN’ [‘MOVE’] for an ME block, cf. Fig. 1C in supplementary material). This was followed by a rest phase of 18–22 s duration. A task run comprised 12 task blocks. Each task block consisted of a 15 s task period indicated by the word ‘START’ and 18–22 s of rest period indicated by the word ‘STOP’. For finger tapping blocks an arrow pointing to the left or the right indicated which hand to use. In whole body movement blocks the arrow was replaced by a human silhouette. Participants were instructed to execute or imagine the movement for as long as the word ‘START’ was on screen and then stop. After six task blocks and between two task runs subjects had 15 s breaks. Participants were instructed to move as little es possible during the whole experiment. The fMRI session was concluded by an anatomical scan.

### Data recording

#### Electromyography (EMG)

Electromyography (EMG) data were recorded from the extensor digitorum communis muscle on both arms. Two electrodes were placed on each arm, resulting in two bipolar channels. In the fNIRS session, the common electrode was attached to the processus styloideus ulnae and in the fMRI session to the left ankle. In the fNIRS session, EMG was recorded with BrainVision Recorder (version 1.21.0303) using a BrainAmp DC Amplifier in combination with a BrainAmp ExG MR (BrainProducts, Gilching, Germany). In the fMRI session an MR-compatible BrainAmp MR in combination with a BrainAmp ExG MR (BrainProducts, Gilching, Germany) and MR-compatible electrodes were used. The sampling rate was 1 kHz with online filtering between 0.1 and 250 Hz.

#### Functional near infrared spectroscopy (fNIRS)

**Figure 4:**
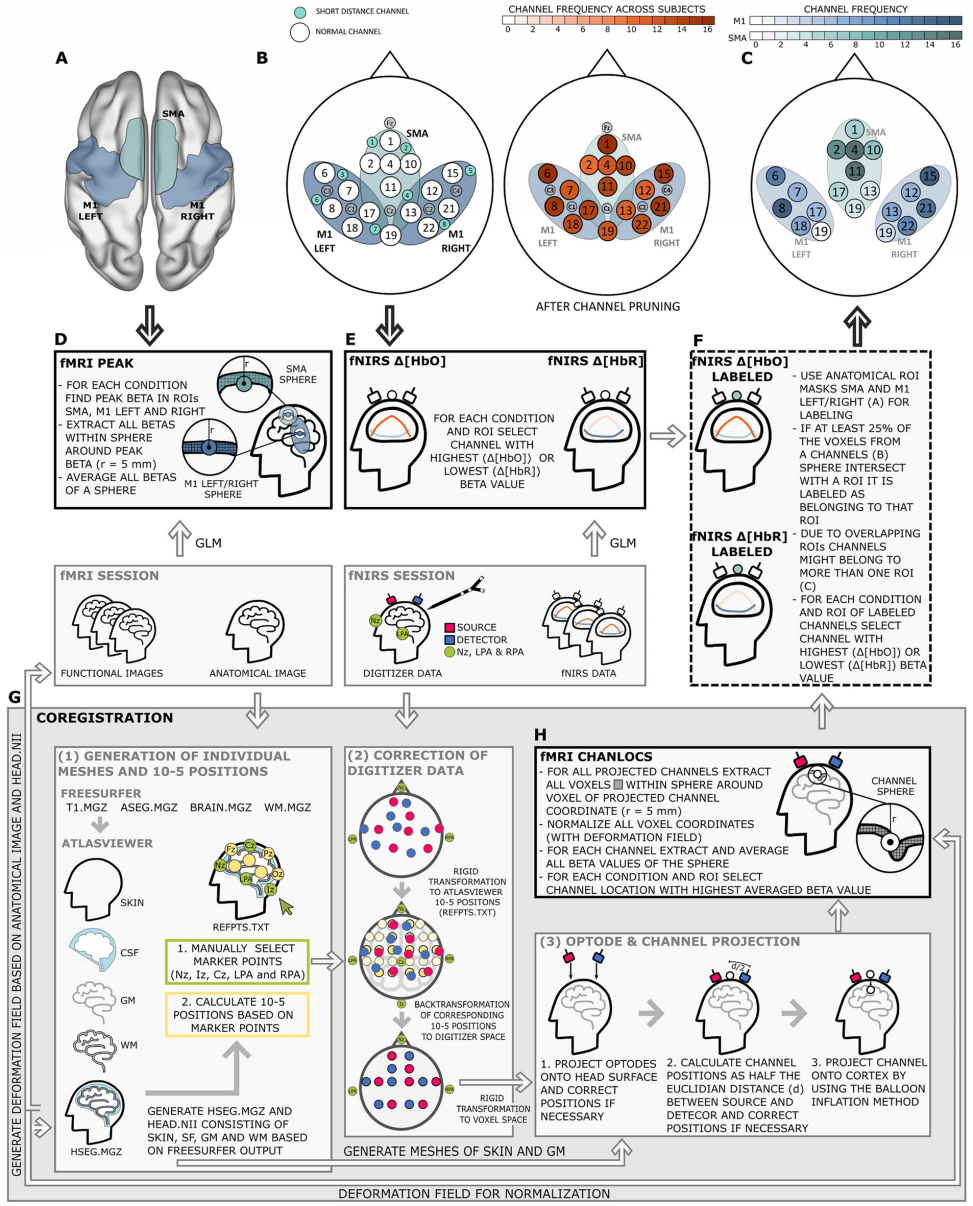
Schematic illustration of **(A)** the ROIs for the fMRI data extraction and **(B)** the channel layout and corresponding fNIRS ROIs approximated by means of the fOLD toolbox^5^ for the fNIRS data extraction. In addition, on the right side of **(B)** the channel frequency across subjects after channel pruning is visualized. For each subject the channel positions and the individual anatomical images were co-registered and channels were labeled as covering one of the fMRI ROIs or not. **(C)** Illustrates the frequency at which channels were labeled as covering an fMRI ROI. For easier comparison channels are overlaid on the fNIRS ROIs. The fNIRS ROIs and the corresponding channels are separately illustrated because some channels were assigned to more than one ROI (e.g., channel 19). The lower part of the figure illustrates all steps taken to extract data types **(D)** fMRI PEAK, **(E)** fNIRS $\Delta [HbO]$ and $\Delta [HbR]$, **(F)** fNIRS $\Delta [HbO]$ and $\Delta [HbR]$ LABELED and **(H)** fMRI CHANLOCS. Boxes representing the final data type are highlighted with solid or dashed black lines. Data types **(D,E)** were derived directly from the functional data sets. For data types **(F,H)** co-registration of MRI and NIRS data was required. The large grey box **(G)** highlights the coregistration steps. In the figure, arrows between and within the smaller boxes illustrate the work flow. Arrows pointing from A to D and from B to E indicate that the areas from which functional data were extracted were restricted by a-priori ROI definitions. The arrow pointing from F to C indicates that **(C)** shows a result of co-registration and of labelling fNIRS channels based on individual anatomy.

FNIRS data were recorded with a NIRScout 816 device (NIRStar 15.2, NIRx Medizintechnik GmbH, Berlin, Germany). The eight LED sources (intensity 5 mW/wavelength) and the eight detectors resulted in a total of 20 channels of which the 16 channels covering both hemispheres of M1 and SMA (approximated with the fOLD toolbox^5^ (v2.2; BrainAtlas: AAL2; Anatomical Landmarks: Supp$\_$Motor$\_$Area$\_$L and Supp$\_$Motor$\_$Area$\_$R; BrainAtlas: Brodmann; Anatomical Landmarks: 4—Primary Motor Cortex; Specificity: 30%) were selected for analysis (cf. Fig. 4B). For the removal of systemic activity artifacts eight short separation detectors were attached to the eight light sources. The NIRS optodes were placed according to the international 10-5 system in a custom-made cap. The Cz position was used as a marker for correctly positioning the cap. If necessary optodes were attached to the cap with spring-loaded grommets (NIRx Medizintechnik GmbH, Berlin, Germany). This helped to reduce optode movement and improved contact between optodes and scalp. The distance between a source and a regular detector was approximately 3 cm, whereas the distance between a source and its short-distance detector was fixed at 0.8 cm. The sources emitted near infrared light at wavelengths 760 nm and 850 nm. Light intensity was sampled with a rate of 7.8125 Hz. For each subject optode positions and fiducial points (Nz, LPA and RPA) were digitized with an optical digitizer (Xensor, ANT Neuro, The Netherlands) at the end of the fNIRS session in order keep to the course of fMRI and fNIRS sessions comparable.

#### Functional magnetic resonance imaging (fMRI)

FMRI data were collected with a 3T whole-body Siemens Magnetom Prisma MRI and a 20-channel head coil (Siemens AG, Erlangen, Germany). Functional images were acquired with an ascending echo-planar imaging (EPI) sequence (voxel size $= 3\times 3\times 3$; TR $=$ 2000 ms, TE $=$ 30 ms, flip angle $=$ 75$^{\circ }$; FoV $=$ $192\times 192$ mm, base resolution $=$ $64\times 64$ voxels; 36 transversal 3 mm slices with a gap of 10% acquired in an interleaved mode; phase coding direction: anterior to posterior). T1-weighted structural images were acquired with a magnetization prepared rapid gradient-echo (MP-RAGE) sequence (voxel size $=$ $0.8\times 0.8\times 0.8$; TR $=$ 2000 ms, TE $=$ 2.07 ms, flip angle $=$ 9$^{\circ }$; FoV $=$ $240\times 240$ mm, base resolution $=$ $320\times 320$ voxels; 224 sagittal 0.75 mm slices with a gap of 50 %; phase coding direction: anterior to posterior).

## Data processing and statistical analysis

### Data preprocessing

#### Electromyography (EMG)

EMG signals were collected in order to control for the absence of voluntary movements within the MI trials. The EMG data of the ME tasks served as training set. A classifier was trained to distinguish movement and no movement trials (Classification Learner App; The MathWorks Inc., Natick, MA, USA). For each session, the training data set comprised 10–12 task phases of finger tapping EMG (one subject stopped typing in two trials of the fNIRS session).

EMG preprocessing was mainly performed by means of the EEGLAB toolbox (v14.2.1b^45^) using MATLAB R2019b (The MathWorks Inc., Natick, MA, USA). For the fMRI session the MRI gradient artifact was removed by an add-on function (pop$\_$fmrib$\_$fastr^46^) of eeglab that uses an average artifact template removal in combination with temporal principal component analysis (PCA). All subsequent preprocessing steps were identical for both sessions and included high-pass FIR filtering (pop$\_$firws function; hamming window) with a cut-off frequency of 20 Hz and a filter order of 330, and wavelet denoising (Wavelet Signal Denoiser toolbox; The MathWorks Inc., Natick, MA, USA) with a Daubechies 4 (db4) wavelet (method: Bayes, threshold rule: soft; noise estimate: level dependent). The data was then epoched [0–15] s around stimulus onset for the training set and [−2.5–20] s around stimulus onset for the test set. For each epoch a root mean square envelope of the rectified signal was calculated (MATLAB function envelope() with a window length of 100 samples) and subsequently cut into 7.5 s segments, resulting in 20 to 24 movement and 20 to 24 no movement segments for each hand in the training set and in a total of 108 segments for the test set (3 MI tasks). For each of these segments, mean, standard deviation, wavelength, maximum value and log detector^47^ served as features for training the classifier based on a linear support vector machine algorithm.

If for a participant more than half of the trials of a single MI task were classified as movement trials the whole data set (fNIRS and fMRI) was excluded. This was the case in four participants (two fNIRS, two fMRI). Two additional subjects were excluded because no EMG analysis was possible for the MRI session due to signal saturation. On average 11.77 ($\pm 0.95$, ranging from 6 to 12 trials) MI trials (MI LEFT: 11.44 ± 1.55; MI RIGHT: 12 ± 0; MI WHOLE BODY: 11.88 $\pm 0.50$) of the fMRI data and 11.40 ($\pm 1.57$, ranging from 6 to 12 trials) MI trials (MI LEFT: 11.38 ± 1.54; MI RIGHT: 11.50 ± 1.51; MI WHOLE BODY: 11.38 $\pm 1.54$) of the fNIRS data remained for analyses.

##### Functional near infrared spectroscopy (fNIRS) Preprocessing

 Signal quality was assessed by means of the qt-nirs toolbox (https://github.com/lpollonini/qt-nirs; q-threshold $=$ 0.65, sci-threshold $=$ 0.6, psp-threshold $=$ 0.1). Based on this, one subject had to be excluded because no channel remained for analysis. Furthermore, only data of those subjects were further processed if at least two channels in each ROI (SMA, M1 LEFT and M1 RIGHT) remained for analysis. This was the case for N $=$ 16 individuals. Overall, on average across all subjects $2.69 \pm 3.05$ channels (range: 0 to 8 channels) out of the 16 channels covering the ROIs were pruned (SMA channels: $1.56 \pm 1.63$; M1 LEFT channels: $0.88 \pm 1.31$; M1 RIGHT channels: $1.19 \pm 1.38$). The frequency of the remaining channels across all subjects is visualized in Fig. 4B. Across all subjects, all eight short distance channels remained for analysis. FNIRS data were analysed with a combination of the NIRS Brain AnalyzIR toolbox^48^ and custom made scripts. Raw data were transformed into optical density changes. Motion artifacts were corrected by means of the Temporal Derivative Distribution Repair (TDDR) approach^49^. In a next step the optical density changes were band-pass filtered with a zero-phase digital FIR filter ([0.01, 0.09]Hz with filter order $=$ 1000 as recommended by Pinti et al.^42^). Data were then converted into hemoglobin concentration changes by means of the modified Beer-Lambert law (partial pathlength factor (PPF) $=$ differential pathlength factor (DPF)*partial volume factor (PVF $=$ 1/60)) using individual age-related DPFs^50^. Finally, systemic artifact correction was applied by means of a short distance channel regression procedure using all eight short distance channels of both $\Delta [HbO]$ and $\Delta [HbR]$ as regressors^11,12^ with an autoregressive iterative least square (AR-ILS) model^51^. Note, that for the correction procedure no task-related regressors were included and therefore, after running the GLM the residuals, which are in this case the cleaned data, were selected for further processing. Details for preprocessing can be found in Table 1 of the supplementary material.

##### General linear model

 The data were analysed with an autoregressive iterative least square (AR-ILS) model^51^. All five tasks, task instructions and breaks as well as their first and second derivatives were modeled with a canonical hemodynamic response function (delay $=$ 6 s). In addition, a constant term modeling the trend was added as additional regressor. If movement was detected in the EMG analysis the corresponding MI task blocks were modeled in an additional regressor and were excluded from the estimation of MI-related activation. Resulting beta values were extracted from the main regressor of each task.

#### Functional magnetic resonance imaging (fMRI)

FMRI data were analysed with the Statistical Parametric Mapping toolbox (SPM12; Wellcome Trust Centre for Neuroimaging, London, UK) in combination with MATLAB 2019a. As recommended by Poldrack et al.^52^ and in order to ensure transparency and replicability, all details for preprocessing can be found in Table 2 of the supplementary material.

##### Preprocessing

 In order to remove movement artifacts, fMRI data were registered to the mean image and resliced. The exported realignment parameters were visually inspected and served as quality control measures. No subject was excluded due to excessive head movement (image-to-image movement < 3 mm). Subsequently the data were coregistered to the realigned mean image and to the canonical single subject T1 image (SPM12) with respect to the functional and anatomical images, respectively. The coregistered data were afterwards segmented, bias corrected and spatially normalized and finally smoothed using a Gaussian kernel (full width half maximum $=$ 8 mm).

##### General linear model

 In the GLM analysis a temporal high-pass filter (128 s) was applied and temporal autocorrelations across scans were modeled with an AR (1) model. The masking threshold was set to zero in order to ensure that the whole cortex was included in the analysis. In spite of this, for one subject relevant voxels were not included in the mask. The subject was therefore excluded from analysis. Head movement parameters, a constant term and additionally to the regressors of the five main tasks, task instruction and break regressors, the first and second derivative were as well modeled within the GLM. For subjects with detected movement in the MI task blocks an additional regressor containing all the contaminated MI trials entered the GLM analysis in order to keep out movement contaminated trials from estimating MI-related activation. Data were then modeled with the canonical hemodynamic response function (delay $=$ 6 s) as basis function.

#### fMRI-fNIRS coregistration

With the coregistration, normalized Montreal Neurological Institute (MNI) coordinates of the fNIRS channels projected to the cortex were obtained. FMRI beta values and the BOLD signal were extracted from these coordinates for comparison with the fNIRS channel data. The coregistration workflow is illustrated in Fig. 4G and explained in detail below.

##### Generation of individual meshes and 10-5 positions

 The first step of coregistration was the generation of individual head and brain meshes (cf. Fig. 4G1). For this, the subject-specific anatomical images were segmented using the ’recon-all’ command of the freesurfer software (version 6^53^). A subset of the resulting freesurfer data (i.e., T1.mgz, brain.mgz, aseg.mgz, wm.mgz) were further processed with the AtlasViewer toolbox (v2.8^6^) in combination with MATLAB 2016a. In AtlasViewer, a nifti (head.nii) and an mgz (hseg.mgz) file were created that contained structural MRI information consisting of head surface, CSF, grey and white matter. The hseg.mgz was used within AtlasViewer to select individual marker points (Cz, LPA, RPA, Nz and Iz) and to automatically generate 10-5 positions based on these marker points. Coordinates of the individual marker points and the 10-5 positions were extracted for further analysis.

##### Correction of digitizer data

 For all following steps custom made scripts were used in combination with MATLAB 2019a. The NIRS optode positions derived from the digitizer data were distorted in many data sets due to difficulties in hardware handling. Therefore, for all subjects, NIRS optode positions were derived from the 10-5 positions generated by AtlasViewer. For this, the MRI fiducials (Nz, LPA and RPA) and with it the 10-5 positions were transformed to the corresponding fNIRS digitized fiducials by means of a rigid transformation^54^ (https://github.com/nghiaho12/rigid_transform_3D). The transformed 10-5 positions were then assigned to the corresponding NIRS optode positions. This procedure resulted in undistorted optode positions (cf. Fig. 4G2).

##### Optode and channel projection

 The structural MRI outputs (hseg.mgz) from AtlasViewer were used to generated head surface and brain (grey matter) meshes (iso2mesh toolbox^55,56^). NIRS optode positions were projected to the head surface with a rigid transformation^54^. More precisely, the undistorted optode positions were transformed to the head surface by using the three fiducial markers of both the MRI and the NIRS coordinate sets. Optode locations that were projected to locations outside of or within the head surface were corrected by taking the closest (outer) head surface point lying on a straight line between the head surface’s center and the projected optode location (cf. Fig. 4G3).

Based on the head surface optode locations, channel positions were calculated as the midpoint of the Euclidian distance between a channels’ source and detector. If necessary, channel positions were corrected in the same way as described for optodes. In order to create fMRI channels, head surface channel positions were then projected onto the cortex mesh by using the balloon inflation method^57^. The average distance between head surface and projected cortex position across subjects and channels was (mean ± SD) (16.88 ± 1.80) mm, ranging from 14.60 to 20.27 mm. Averaged distances across subjects are illustrated in Fig. 3 of the supplementary material.

#### Voxel extraction and MNI coordinates of channel positions

For each fMRI channel, the cortex voxel resulting from the previous step served as the center of a sphere with a radius of $r = 5$ mm. The voxel coordinates within each of the spheres were extracted for further processing^27^. Across subjects and channels, this procedure resulted in an average number of ($209.94 \pm 13.81$) voxels per fMRI channel, ranging from a minimum of 185.94 to a maximum of 240.74 voxels.

As a final step, the extracted voxel coordinates were transformed into normalized MNI coordinates. Therefore, individual deformation fields were generated by employing the Deformations Utility of SPM12, using the deformation generated by SPM12 and the inverse of the normalized anatomical image in composition with the nifti file from the AtlasViewer output (head.nii). The resulting 5-dimensional deformation field was used to transform the voxel space coordinates to normalized MNI coordinates.

#### Voxel extraction based on local activation peaks

In addition to voxel extraction based on the projected channel positions, voxels were extracted based on activation peaks within anatomical masks. Three anatomical masks were generated based on the AAL atlas of the wfupickatlas toolbox in SPM12 (v2.4^58,59^). Masks comprised left and right M1 (ROIs M1 LEFT and M1 RIGHT; 3D dilatation = 1) represented by the precentral gyrus, and the bilateral supplementary motor area (ROI SMA). ROIs are illustrated in Fig. 4A. Within each ROI and separately for each task, the maximum beta value was determined and the normalized MNI coordinates of the voxels of a sphere ($r = 5$ mm) around the position of this value were extracted.

### Beta values and time series

Beta values were extracted for fMRI channel voxel spheres, for activation peak voxel spheres, and for the regular fNIRS channels. The voxel sphere betas were averaged within a sphere. This resulted in one beta value per fNIRS channel, one beta value per fMRI channel, and one beta value per fMRI ROI for each subject and task. Furthermore, for all subjects and tasks time series data were extracted. FMRI BOLD signals and fNIRS concentration changes were epoched ([−2.5 to 20] s around stimulus onset) and baseline corrected ([−2 to 0] s around stimulus onset). For the fMRI data, epochs were first averaged for each voxel and then averaged across all voxels of a sphere, resulting in one grand average BOLD signal per channel or ROI. For the fNIRS data epoched time series was averaged channel wise, resulting in one grand average signal per channel.

### Individual anatomical information: labeled channels

To investigate whether guiding fNIRS channel selection by individual anatomical images improves task sensitivity and spatial specificity, fNIRS channels were labeled regarding their ROI affiliation. That is, an fNIRS channel was labeled as belonging to one of the three ROIs (M1 LEFT, M1 RIGHT and/or SMA) if at least $25\%$ of the sphere voxels of the corresponding fMRI channel were part of the ROI mask. All 16 channels passed through this process for each ROI. As a result a channel at the border of several ROIs can belong to each of these ROIs.

For M1 LEFT, an average of (± SD) (3.00 ± 0.89) channels (ranging from 2 to 5 channels) was assigned to ROI M1 LEFT by the labeling process. Regarding ROI M1 RIGHT, on average (± SD) (3.56 ± 1.09) channels (ranging from 2 to 5 channels) were labeled as belonging to the ROI. For ROI SMA on average (± SD) (4.25 ± 0.68) channels (ranging from 3 to 6 channels) were labeled as ROI channels. The resulting channel frequencies of this labeling process is shown in Fig. 4C.

### Data types

Analyses comprised four functional data types: (1) fMRI PEAK data (derived from averaged betas of the sphere around the beta peak within an ROI; 5 tasks $\times$ 3 ROIs per subject; cf. Fig. 4D), (2) fMRI CHANLOCS data (derived from the averaged betas of the sphere around each projected channel location; 5 tasks $\times$ 16 channel spheres per subject and ROI; cf. Fig. 4H), (3) fNIRS $\Delta [HbO]$, and (4) fNIRS $\Delta [HbR]$ data (5 tasks $\times$ 16 channels per subject and ROI; cf. Fig. 4E). For the fNIRS data, analyses were also performed on individually labeled channel data only (cf. section “Individual anatomical information: labeled channels”). These subsets are referred to as fNIRS $\Delta [HbO]$ LABELED and fNIRS $\Delta [HbR]$ LABELED (5 tasks $\times$ 16 channels per subject and ROI; cf. Fig. 4F).

### Statistical analyses

Statistical analyses were performed with R (v4.0.2 ’Taking Off Again’^60^) in RStudio (v1.3.1093 ‘Apricot Nasturtium’^61^) and JASP (v0.15^62^). In order to correct for violations of sphericity (repeated measures analysis of variance; rmANOVA) the Greenhouse-Geisser correction method was used. As post hoc tests paired Student’s t-tests or pairwise t-tests were applied. In order to correct for multiple comparisons false discovery rate^63^ correction was used and either applied within a ROI (i.e., M1 or SMA in the following paragraphs *Within subjects: time series correlation* and *Within subjects: repeated measures correlation*), across conditions irrespective of ROI (i.e., paragraph *Between subjects: topographical similarity*) or within an statistical test (i.e., post hoc tests after ANOVA in paragraph *Between subjects: task-related activation patterns*). Additionally, test specific effect sizes (Cohen’s d or generalized $\eta _{G}^{2}$) and $95\%$ CI are reported.

#### Spatial specificity

Spatial specificity describes how well a signal is defined in space. For non-invasive functional neuroimaging fMRI is considered the gold standard for spatial specificity. In the present study spatial specificity of fNIRS data was assessed by considering topographical similarity with fMRI CHANLOCS data and channel-specific time series correlations with fMRI PEAK data.

##### Between subjects: topographical similarity

 fNIRS channel beta values were compared to fMRI CHANLOCS beta values on a between subjects level. For each data type (fMRI CHANLOCS, fNIRS $\Delta [HbO]$, fNIRS $\Delta [HbR]$) and task separately, betas were averaged for each channel across subjects. This resulted in one topographic beta map for each task and data type. The topographic map of data type fMRI CHANLOCS was considered the gold standard. To examine the similarity between beta maps Spearman correlations were conducted. It was expected that the fNIRS beta maps show strong positive ($\Delta [HbO]$) or negative ($\Delta [HbR]$) relationships with the fMRI CHANLOCS beta maps. Furthermore, strong negative relationships between fNIRS $\Delta [HbO]$ and fNIRS $\Delta [HbR]$ beta maps were anticipated. Map correlations were expected to be strongest for ME tasks, but also for the MI tasks significant correlations were assumed.

##### Within subjects: time series correlation

For each individual subject, ROI and task, the fMRI PEAK time series data were extracted and averaged across all voxels obtained by the procedure described in section *Voxel extraction based on local activation peaks*. For data types fMRI CHANLOCS, $\Delta [HbO]$ and $\Delta [HbR]$ as well as $\Delta [HbO]$ LABELED and $\Delta [HbR]$ LABELED, the channel with the strongest beta value of each task and ROI was selected for each subject individually. For fNIRS data types the time series signal of the respective channel was extracted for this analysis. For fMRI CHANLOCS, all time series of the voxels within the selected channel sphere were extracted and averaged before entering the analysis. This approach allowed us to test the correspondence between the signal time series at the individual fMRI peak location, which is unrestricted by channel location, and the individual fNIRS or fMRI CHANLOCS peak location, i.e., the channel associated with the largest signal for a given task within a ROI.

Single subject fMRI PEAK time series data were correlated with the single subject time series data of the individually selected channels of fMRI CHANLOCS, fNIRS $\Delta [HbO]$ or fNIRS $\Delta [HbR]$ time series data and the LABELED versions of the fNIRS data. Correlations were run separately for each task. Resulting Spearman correlation coefficients were Fisher z-transformed and one sample t-tests were conducted for each task and each fMRI PEAK—channel data pair. For the ROIs M1 LEFT and M1 RIGHT only ME tasks (ME LEFT and ME RIGHT) were included in the analysis. For the ROI SMA all tasks were included. It was expected that fMRI PEAK shows the strongest positive relationship with fMRI CHANLOCS data, followed by a reduced but still significant (positive or negative) relationships with fNIRS data types. Stronger correlations for labeled compared to unlabeled fNIRS data types would indicate a benefit from individual MRI-fNIRS coregistration.

#### Task sensitivity

Task sensitivity describes how well signal changes are driven by task changes. For judging task sensitivity, the pattern of fMRI PEAK signal changes in consequence to task was considered as representative for all other data types. The latter were regarded as task sensitive when their pattern of task-related signal changes was comparable to the pattern of fMRI PEAK changes.

For both task sensitivity analyses, for data types fMRI CHANLOCS, fNIRS $\Delta [HbO]$, fNIRS $\Delta [HbR]$, fNIRS $\Delta [HbO]$ LABELED, and fNIRS $\Delta [HbR]$ LABELED the channel with the strongest beta value was selected. This was done separately for each subject, task (ME LEFT, ME RIGHT, MI LEFT, MI RIGHT and MI WHOLE BODY) and ROI, and thus resulted, for each data type, in five task-related beta values per ROI. An overview of the channel selection frequency for each data type, ROI and task is given in supplementary material Fig. 2A,B.

Due to the high between-subjects variability of fNIRS beta values, individual min–max normalization (cf. Eq. 1) was performed based on the five task-related beta values separately for each data type.

1 $$\begin{aligned} x_{norm} = \frac{x - min(x)}{max(x) - min(x)} \end{aligned}$$

In Eq. (1) *x* represents the vector of the five task-related beta values per ROI for a single subject. For fNIRS $\Delta [HbR]$ data, the absolute value of each beta value was used. After min-max normalization the five betas range from 0 (lowest beta) to 1 (highest beta).

##### Between subjects

Task-related activation patterns Regarding the M1 ROI, for each data type (fMRI PEAK, fMRI CHANLOCS, fNIRS $\Delta [HbO]$ and fNIRS $\Delta [HbR]$ [and LABELED versions]) a $2\times 2$ rmANOVA with the within subjects factors task (ME LEFT, ME RIGHT) and hemisphere (LEFT, RIGHT) was conducted with the min–max normalized (over all five tasks) beta values as dependent variables. For the SMA ROI, a one factorial rmANOVA with the within subjects factor task (ME LEFT, ME RIGHT, MI LEFT, MI RIGHT, MI WHOLE BODY) was implemented with the min-max normalized beta values as dependent variables. The overall expectation was to see comparable rankings of signal changes related to tasks for fMRI PEAK and each of the other data types. Regarding the M1 ROIs, the predicted pattern was that of a lateralisation of activity, that is, a significantly stronger activation in the hemisphere contralateral to the executing hand. For ROI SMA we predicted higher activation for the ME compared to the MI tasks, without specific predictions for the different MI tasks.

##### Within subjects

Repeated measures correlation Repeated measures correlations (’rmcorr’ package^33^) were conducted with min–max normalized betas of data type fMRI PEAK and each of the other data type (fMRI CHANLOCS, fNIRS $\Delta [HbO]$ and fNIRS $\Delta [HbR]$ [and LABELED versions]). Correlations were run for each ROI (M1 LEFT, M1 RIGHT and SMA). A strong positive relationship represented by a high repeated measure correlation coefficient between fMRI PEAK and another data type would be indicative of task sensitivity within subjects.

## Supplementary Information

Supplementary Information.

## Data Availability

The data sets generated and/or analysed during the current study are not publicly available due to data protection issues but are available from the corresponding author on reasonable request.
