# Supplementary material for: fMRI-based validation of continuous-wave fNIRS of supplementary motor area activation during motor execution and motor imagery
Source: Sci Rep. 2022 Mar 4;12:3570. doi: 10.1038/s41598-022-06519-7 (PMC8897516; doi:10.1038/s41598-022-06519-7)
Supplement: Supplementary file 1 — Supplementary Information. [file 41598_2022_6519_MOESM1_ESM.pdf]

Supplementary Material

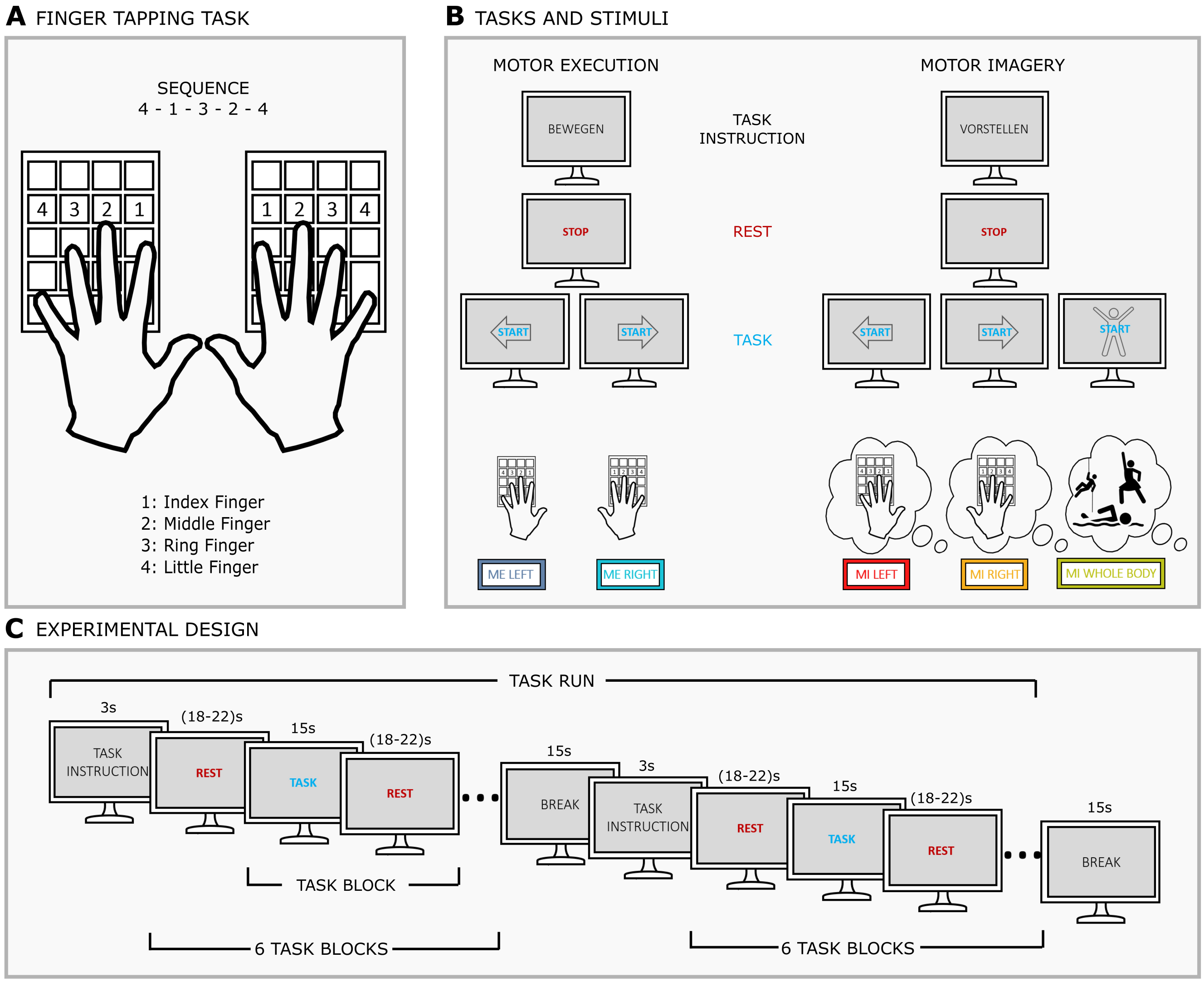

Figure 1. Schematic illustration of (A) the finger tapping task, (B) the stimuli of the respective tasks and (C) of the experimental design. (A) In order to type the sequence of the finger tapping task, the subjects were instructed that each finger of each hand corresponds to a specific number which should be used to press the respective key on a number pad. (B) tasks were either motor execution of left or right hand finger tapping (bottom left) or motor imagery of this task or a whole body movement (bottom right). The stimuli are shown in the upper part. At the beginning of each task run and after each break a task instruction indicated whether the following task is ME ('BEWEGEN') or MI ('VORSTELLEN'). The stimuli displayed whether the task was left or the right hand (ME and MI) or the whole body movement (MI only). (C) Each task run started with the task instruction (3s) and a rest period of 18-22s and consisted of 12 task blocks of 15s task period followed by 18-22s of rest period. After 6 task blocks a break of 15s duration was integrated.

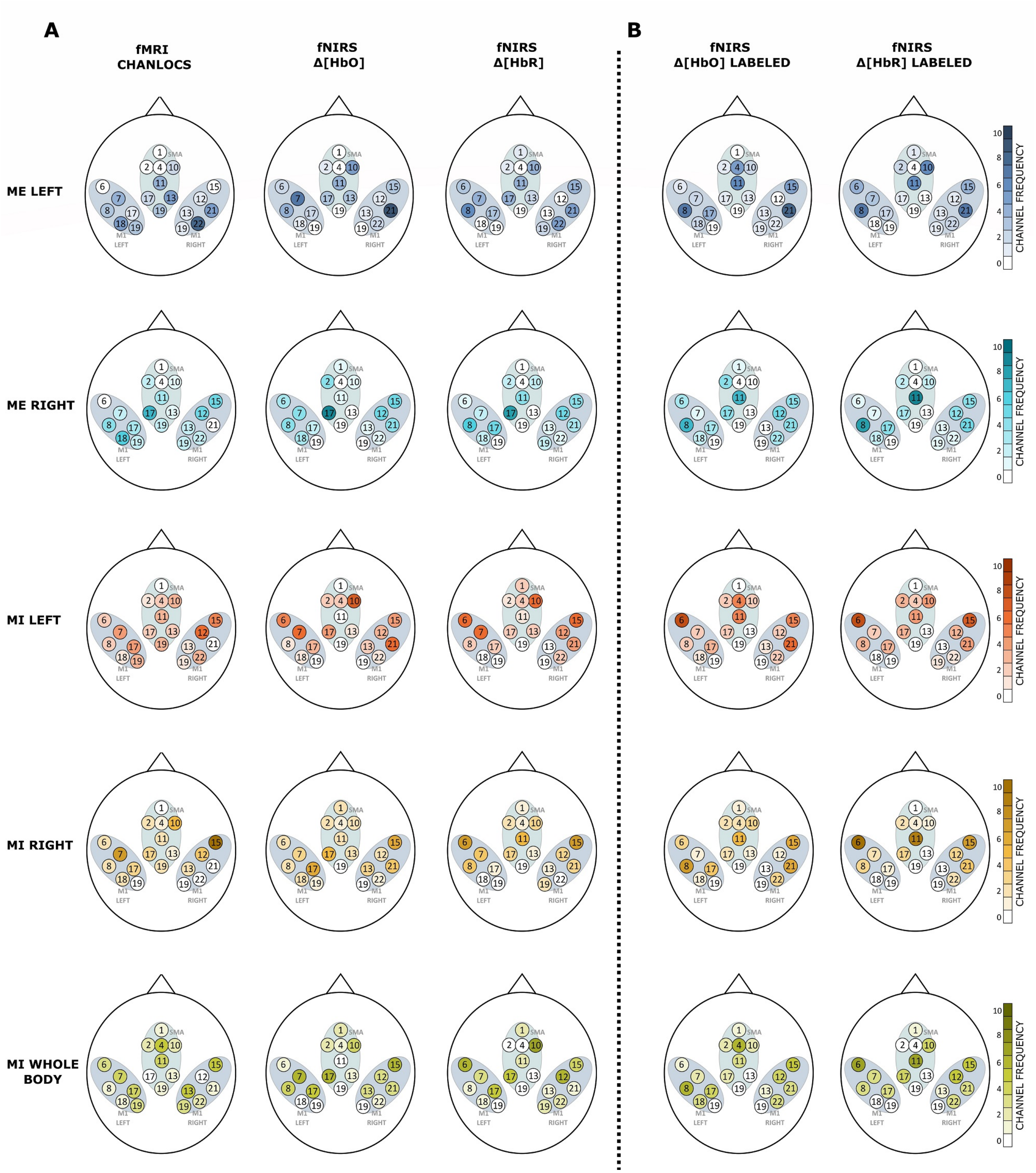

Figure 2. Channel selection frequency for data types (columns) and tasks (rows) split by ROI. Illustrated is how often a channel was selected across subjects for an ROI based on its beta value. Note that a subset of channels (13, 17, 19) belonged to more than one ROI and were therefore considered for selection several times. (A) Channel selection frequencies for data types fMRI CHANLOCS, fNIRS $\Delta[HbO]$ and fNIRS $\Delta[HbR]$. (B) Channle selection frequencies for data types fNIRS $\Delta[HbO]$ LABELED and fNIRS $\Delta[HbR]$ LABELED. Color bars at the right end of each row apply for all five data types.

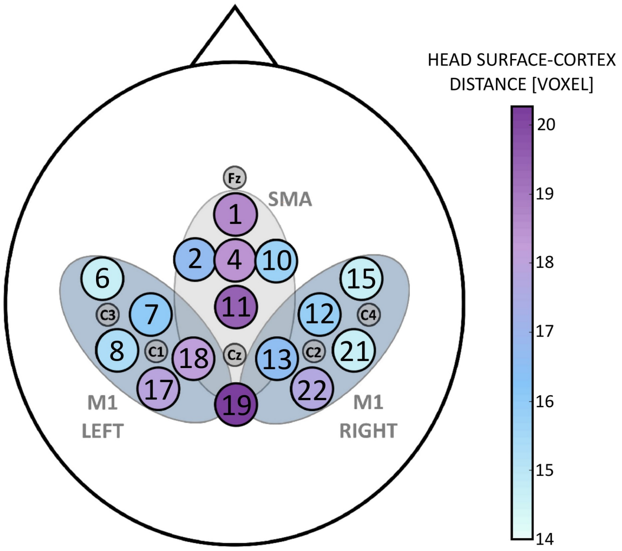

Figure 3. Visualisation of the distance between fNIRS channel position on the head surface and its projection to the cortex in voxel space. Each channels' distance incorporates the average distance across subjects. Darker colors indicate longer distances and brighter colors shorter distances. Voxel extraction is based on the head.nii file generated by AtlasViewer, therefore, a voxel has the size $1 \times 1 \times 1$mm.

Table 1. Table containg the fNIRS preprocessing steps in the correct order with all necessary inputs and variables.

**fNIRS Preprocessing Pipeline**	
(1) Quality check using qt-nirs toolbox	job = nirs.modules.QT;(qt-toolbox in NIRS toolbox)<br>job.sciThreshold = 0.6;<br>job.qThreshold = 0.65;
(2) Convert raw intensity data to changes of optical density	job = nirs.modules.OpticalDensity; (NIRS toolbox)
(3) TDDR motion correction	job = nirs.modules.TDDR; (NIRS toolbox)
(4) Apply band-pass filter	lpf_cutoff = 0.09;<br>hpf_cutoff = 0.01;<br>order = 1000;<br><br>bpFilt = designfilt('bandpassfir', 'FilterOrder', order, ...<br>'CutoffFrequency1', hpf_cutoff, 'CutoffFrequency2', lpf_cutoff, ...<br>'SampleRate', dat.Fs);<br>bp_data = filtfilt(bpFilt, dat.data);<br>dat.data = bp_data;
(5) Channel Pruning: replace channels with bad quality detected in (1) with NaNs	
(6) Convert changes in optical density into hemoglobin concentration changes using mBLL	PVF = 1/60;<br>DPF = CalcDPF(age, [760 850]); (function from Scholkmann et al., 2013)<br>job = nirs.modules.BeerLambertLaw; (NIRS toolbox)<br>job.PPF = DPF.*PVF;
(7) Apply systemic artifact correction using GLM in combination with all short distance channels; extract residuals	

Table 2. Table containg the SPM12 fMRI preprocessing steps in the correct order with all necessary inputs and variables in order to run it in a SPM12 batch file.

**SPM12 Preprocessing Pipeline**	
**(1) Realign: Estimate & Reslice**	
Data	
. Session	Functional Images
Estimation Options	
. Quality	1
. Separation	4
. Smoothing (FWHM)	5
. Num Passes	Register to mean
. Interpolation	2nd Degree B-Spline
. Wrapping	Wrap Y
. Weighting	0 files

Reslice Options	
. Resliced Images	Mean Image Only
. Interpolation	4th Degree B-Spline
. Wrapping	Wrap Y
. Masking	Mask images
. Filename Prefix	r
**(2) Coregister: Estimate**	
Reference Image	DEP Realign: Estimate & Reslice: Mean Image
Source Image	Anatomical Image (T1)
Other Images	-
Estimation Options	
. Objective Function	Normalised Mutual Information
. Separation	[4 2]
. Tolerances	[0.02 0.02 0.02 0.001 0.001 0.001 0.01 0.01 0.01 0.001 0.001 0.001]
. Histogram Smoothing	[7 7]
**(3) Coregister: Estimate**	
Reference Image	...\spm12\canonical\single_subj_T1.nii, 1
Source Image	DEP Coregister: Estimate: Coregistered Images
	DEP
Other Images	Realign: Estimate & Reslice: Realigned Images (Sess 1)
	Realign: Estimate & Reslice: Mean Image
Estimation Options	
. Objective Function	Normalised Mutual Information
. Separation	[4 2]
. Tolerances	[0.02 0.02 0.02 0.001 0.001 0.001 0.01 0.01 0.01 0.001 0.001 0.001]
. Histogram Smoothing	[7 7]
**(4) Segment**	
Data	
. Channel	
. . Volumes	DEP Coregister: Estimate: Coregistered Images
. . Bias regularisation	light regularisation (0.001)
. . Bias FWHM	60 mm cutoff
. . Save Bias Corrected	Save Bias Corrected
Tissues	
. Tissue	
. . Tissue probability map	...\spm12\tpm\TPM.nii, 1
. . Num. Gaussians	1
. . Native Tissue	Native Space
. . Warped Tissue	None
. Tissue	
. . Tissue probability map	...\spm12\tpm\TPM.nii, 2
. . Num. Gaussians	1
. . Native Tissue	Native Space
. . Warped Tissue	None
. Tissue	
. . Tissue probability map	...\spm12\tpm\TPM.nii, 3
. . Num. Gaussians	2
. . Native Tissue	Native Space
. . Warped Tissue	None
. Tissue	
. . Tissue probability map	...\spm12\tpm\TPM.nii, 4
. . Num. Gaussians	3
. . Native Tissue	Native Space
. . Warped Tissue	None
. Tissue	
. . Tissue probability map	...\spm12\tpm\TPM.nii, 5
. . Num. Gaussians	4
. . Native Tissue	Native Space
. . Warped Tissue	None
. Tissue	
. . Tissue probability map	...\spm12\tpm\TPM.nii, 6
. . Num. Gaussians	2
. . Native Tissue	None
. . Warped Tissue	None
Warping & MRF	
. MRF Parameter	1
. Clean Up	Light Clean
. Warping Regularisation	[0 0.001 0.5 0.05 0.2]
. Affine Regularisation	ICBM space template - European brains

. Smoothness	0
. Sampling distance	3
. Deformation Fields	Forward
**(5) Normalise: Write**	
Data	
. Subject	
. . Deformation Field	DEP Segment: Forward Deformations<br>DEP
. . Images to Write	Realign: Estimate & Reslice: Realigned Images (Sess 1)<br>Realign: Estimate & Reslice: Mean Image
Writing Options	
. Bounding box	[-78 -112 -70; 78 76 85]
. Voxel sizes	[2 2 2]
. Interpolation	4th Degree B-Spline
. Filename Prefix	w
**(6) Normalise: Write**	
Data	
. Subject	
. . Deformation Field	DEP Segment: Forward Deformations<br>DEP
. . Images to Write	Coregister: Estimate: Coregistered Images<br>Segment: Bias Corrected (1)
Writing Options	
. Bounding box	[-78 -112 -70; 78 76 85]
. Voxel sizes	[1 1 1]
. Interpolation	4th Degree B-Spline
. Filename Prefix	w
**(7) Smooth**	
Images to smooth	DEP Normalise: Write: Normalised Images (Subj 1)
FWHM	[8 8 8]
Data Type	SAME
Implicit Masking	No
Filename Prefix	s
